# Genetic diversity and signatures of selection in various goat breeds revealed by genome-wide SNP markers

**DOI:** 10.1186/s12864-017-3610-0

**Published:** 2017-03-14

**Authors:** Luiz F. Brito, James W. Kijas, Ricardo V. Ventura, Mehdi Sargolzaei, Laercio R. Porto-Neto, Angela Cánovas, Zeny Feng, Mohsen Jafarikia, Flávio S. Schenkel

**Affiliations:** 1 Centre for Genetic Improvement of Livestock, University of Guelph, Guelph, Ontario Canada; 2 CSIRO Agriculture & Food, Brisbane, Queensland Australia; 3 Beef Improvement Opportunities, Guelph, Ontario Canada; 4 The Semex Alliance, Guelph, Ontario Canada; 5 Department of Mathematics and Statistics, University of Guelph, Guelph, Ontario Canada; 6 Canadian Centre for Swine Improvement Inc., Ottawa, Ontario Canada

**Keywords:** *Capra hircus*, F-statistics, hapFLK, Selective sweep, SNP

## Abstract

**Background:**

The detection of signatures of selection has the potential to elucidate the identities of genes and mutations associated with phenotypic traits important for livestock species. It is also very relevant to investigate the levels of genetic diversity of a population, as genetic diversity represents the raw material essential for breeding and has practical implications for implementation of genomic selection. A total of 1151 animals from nine goat populations selected for different breeding goals and genotyped with the Illumina Goat 50K single nucleotide polymorphisms (SNP) Beadchip were included in this investigation.

**Results:**

The proportion of polymorphic SNPs ranged from 0.902 (Nubian) to 0.995 (Rangeland). The overall mean H_O_ and H_E_ was 0.374 ± 0.021 and 0.369 ± 0.023, respectively. The average pairwise genetic distance (D) ranged from 0.263 (Toggenburg) to 0.323 (Rangeland). The overall average for the inbreeding measures F_EH_, F_VR_, F_LEUT_, F_ROH_ and F_PED_ was 0.129, −0.012, −0.010, 0.038 and 0.030, respectively. Several regions located on 19 chromosomes were potentially under selection in at least one of the goat breeds. The genomic population tree constructed using all SNPs differentiated breeds based on selection purpose, while genomic population tree built using only SNPs in the most significant region showed a great differentiation between LaMancha and the other breeds. We hypothesized that this region is related to ear morphogenesis. Furthermore, we identified genes potentially related to reproduction traits, adult body mass, efficiency of food conversion, abdominal fat deposition, conformation traits, liver fat metabolism, milk fatty acids, somatic cells score, milk protein, thermo-tolerance and ear morphogenesis.

**Conclusions:**

In general, moderate to high levels of genetic variability were observed for all the breeds and a characterization of runs of homozygosity gave insights into the breeds’ development history. The information reported here will be useful for the implementation of genomic selection and other genomic studies in goats. We also identified various genome regions under positive selection using smoothed F_ST_ and hapFLK statistics and suggested genes, which are potentially under selection. These results can now provide a foundation to formulate biological hypotheses related to selection processes in goats.

**Electronic supplementary material:**

The online version of this article (doi:10.1186/s12864-017-3610-0) contains supplementary material, which is available to authorized users.

## Background

Natural selection plays a very important role on selecting the individuals that are more adapted to new environmental conditions. Besides natural selection, artificial selection has been widely applied to livestock species in order to achieve more desirable/profitable phenotypes. For instance, goats (*Capra hircus*) have been selected since domestication, which occurred around 10,000 years ago [1, 2]. This process of selection resulted in divergent breeds that are specialized for either milk, fiber or meat production or raised as dual-purpose breeds in different regions of the globe. Natural and artificial selection strategies are likely to impose pressure on specific genome regions that control these traits (i.e. milk, meat and fiber) as well as other important characteristics such as adaptation to different environments, reproduction, body conformation, behavior and resistance to diseases and parasites. The unique genetic patterns left behind in the genome of individuals under natural and/or artificial selection is defined as signatures of selection, which are usually regions of the genome that harbor functionally important sequence variants [3]. The detection of signatures of selection is a relevant topic since it has the potential to elucidate the identities of genes and mutations associated with phenotypic traits even if they are no longer segregating within any of the populations of interest and does not necessarily require phenotypes measures. Furthermore, this knowledge is important in order to better understand the evolution process and the mechanisms that underlie traits that have been exposed to intensive natural and artificial selection. Therefore, we can make use of this information to design and/or update breeding and conservation programs worldwide.

Comparison of goat breeds reveals a large phenotypic variation, however there is still a lack of knowledge concerning the genomic variation that contributes to breeds which have different morphological attributes. The majority of caprine population genetics studies have been limited to a few dozen of markers (i.e., microsatellites) [4, 5]. Recent advances in genomic technologies resulting in the availability of the Illumina Goat 50K SNP BeadChip [6] have offered the opportunity to search for genomic regions that may have undergone selection. Such studies in cattle [7–9], sheep [10, 11], chickens [12] and pigs [13, 14] have each identified genes that have undergone positive selection and are likely to contribute directly to phenotypic variation. However, in goats there are only a few studies using the SNP arrays and most of them focused on local breeds (e.g. Italian [15] and Moroccan breeds [4]). It highlights the need to investigate signatures of selection in breeds that are more common worldwide (e.g. Alpine, Boer, Cashmere, and Saanen) and representing all major breeding goals to make a broad assessment of the effects of selection history in goats.

One of the most popular statistical approaches to detect signatures of selection is the calculation of the fixation index (F_ST_) [16], which is based on the measure of population differentiation due to locus-specific allele frequencies between populations. In other words, F_ST_ test detects highly differentiated alleles, where positive selection in a given genome region causes exaggerated frequency differences between populations. High F_ST_ values indicate local positive adaptation while low F_ST_ values suggest negative or neutral selection. Despite its popularity, as discussed in Fariello et al. [17], F_ST_ statistics may identify a large number of false positives/negatives when applied to hierarchically structured data sets. In addition, the heterogeneity of effective population size (N_e_) among breeds can potentially contribute to large locus-specific F_ST_ values among breed groups [18]. Using the same dataset, Brito et al. [19] reported a variation in N_e_ among the breeds included in this investigation. Therefore, the approach named hapFLK, proposed by Fariello et al. [20] and based on haplotype differentiation between populations, seems like another reasonable alternative to confirm or identify signatures of selection in goat populations.

Selection process may give rise to high levels of homozygosity, also called runs of homozygosity (ROH) [21], that result from parents transmitting identical haplotypes to their offspring. Some studies have also used this information as a measure of inbreeding [22, 23]. However, to date, the extent of ROH across the genome in various goat breeds remained unexplored. Genetic diversity represents the raw material essential for evolution and breeding as it provides the substrate for natural and artificial selection [3]. This makes it important to document the relative levels of genetic diversity within and between populations using metrics such as inbreeding, heterozygosity, average minor allele frequency, proportion of polymorphic SNPs. These metrics also inform breeding and conservation programs to effectively improve the levels of production and reproduction, management and conservation of genetic resources.

The objectives of this study were: 1) to present a comprehensive genome-wide analysis of genetic diversity of a variety of the worldwide most common goat breeds; 2) to detect signatures of selection using a 50K SNP chip using different methodologies and the most common breeds raised for fiber, meat and/or milk production and geographically distinct populations of the same breed (i.e. Boer); 3) to provide, for the first time, a comprehensive characterization of ROH in the goat genome using a collection of diverse breeds; and 4) to examine potential biological functions and metabolic pathways of the genes in the identified regions of selection signatures.

## Methods

### Animals and genotypes

A total of 1151 animals from nine goat populations were included in this study. The dataset used here has been previously described [19, 24]. In brief, there were between 48 (Cashmere) and 403 (Alpine) animals genotyped per breed. Two sources of genotypes were included: i) a set of 976 Canadian goats from six breeds (Alpine, Boer, LaMancha, Nubian, Saanen and Toggenburg) and ii) 175 Australian goats from three breeds (Boer, Cashmere and Rangeland). These animals can be grouped in four categories based on main selection objective: milk (Saanen, Alpine, LaMancha and Toggenburg), meat (Australian and Canadian Boer populations and Rangeland), fiber (Cashmere) and dual-purpose (Nubian).

All the animals were genotyped with the Illumina Goat 50K SNP BeadChip [6] containing 53,347 single nucleotide polymorphisms (SNPs). SNP filtering and quality control conducted on the Australian populations resulted in analysis of a final marker set containing 52,088 loci [24]. The Canadian and Australian datasets were merged and only the 52,088 SNPs present in both datasets were kept for further analysis. SNPs with minor allele frequency (MAF) lower than 0.01, call rate lower than 95%, SNPs located on the X chromosome or without known position in the genome were excluded from the analysis. The number of SNPs remaining after the quality control was 48,417 out of 52,088 SNPs.

### Genetic diversity metrics

Various metrics were used to estimate levels of within-breed genetic diversity (Table 1). The different number of samples per population/breed could bias the analysis. Therefore, we performed the analysis using either 48 randomly selected animals (smallest sample size) from each breed or all the genotypes available. The results were then compared.

**Table 1:** Summary of genotyped animals and genetic diversity compared between nine goat populations

Breed	Alpine	Boer	Boer	Cashmere	LaMancha	Nubian	Rangeland	Saanen	Toggenburg
Origin	Canada	Australia	Canada	Australia	Canada	Canada	Australia	Canada	Canada
Abbreviation	AL	BA	BC	CA	LA	NU	RA	SA	TO
Sample size	403	61	67	48	81	54	66	318	53
Purpose	Milk	Meat	Meat	Fiber	Milk	Milk/Meat	Meat	Milk	Milk
P_N_^a^	0.946	0.969	0.924	0.981	0.939	0.902	0.995	0.945	0.911
H_O_^a^	0.385	0.365	0.363	0.384	0.384	0.338	0.413	0.379	0.353
H_E_^a^	0.388	0.356	0.357	0.372	0.382	0.335	0.411	0.382	0.336
D_ST_	0.307	0.281	0.284	0.293	0.303	0.269	0.323	0.302	0.263
F_EH_ ± SD	0.103 ± 0.058	0.141 ± 0.043	0.156 ± 0.048	0.104 ± 0.044	0.108 ± 0.046	0.214 ± 0.051	0.039 ± 0.036	0.117 ± 0.056	0.179 ± 0.055
F_VR_ ± SD	0.006 ± 0.063	−0.029 ± 0.065	−0.014 ± 0.064	−0.027 ± 0.053	−0.001 ± 0.079	−0.004 ± 0.082	−0.001 ± 0.033	0.005 ± 0.090	−0.041 ± 0.223
F_LEUT_ ± SD	0.006 ± 0.093	−0.028 ± 0.087	−0.014 ± 0.105	−0.027 ± 0.080	0.000 ± 0.134	−0.005 ± 0.146	0.000 ± 0.034	0.006 ± 0.138	−0.027 ± 0.373
F_ROH_ ± SD	0.031 ± 0.019	0.047 ± 0.015	0.057 ± 0.016	0.021 ± 0.009	0.039 ± 0.018	0.057 ± 0.018	0.009 ± 0.009	0.033 ± 0.019	0.046 ± 0.018
F_PED_ ± SD	0.021 ± 0.040	NA	0.002 ± 0.016	NA	0.044 ± 0.050	0.017 ± 0.034	NA	0.040 ± 0.042	0.054 ± 0.053

*P*_*N*_ proportion of polymorphic SNPs, *H*_*E*_ *and H*_*O*_ expected and observed heterozygosity, respectively, *D*_*ST*_ average pairwise genetic distance, *SD* standard deviation, *NA* not available, *F*_*EH*_ *, F*_*VR*_ *, F*_*LEUT*_ *, F*_*ROH*_ *and F*_*PED*_ inbreeding coefficients based on excess of homozygosity, VanRaden, Leutenneger, runs of homozygosity and pedigree, respectively

^a^estimates for the three Australian breeds were previously reported by Kijas et al. [24] using the same dataset

#### Heterozygosity

The observed heterozygosity (H_O_) per animal, within breed, was calculated, based on markers which passed the quality control, and compared to the expected heterozygosity under Hardy Weinberg Equilibrium (H_E_). H_O_ was calculated as the number of heterozygotes divided by the total number of genotypes. The estimates were calculated using the –*hardy* flag in PLINK [25] using default settings.

#### Proportion of polymorphic SNPs (P_N_) and average minor allele frequency (MAF)

P_N_ gives the fraction of total SNPs that displayed both alleles within each population. P_N_ was calculated as the proportion of SNPs with MAF greater than 1% within each breed. Both calculations were done after the genotyping quality control. MAF is the frequency estimate of the least common allele per breed.

#### Average pairwise genetic distance (D)

The average pairwise genetic distance separating individuals within each population was calculated in PLINK [25]. Higher values indicate elevated genetic distance between individuals. The average proportion of alleles shared between two individuals was calculated as D_ST_ by PLINK [25]: ${D}_{ST}=\frac{IBS2+0.5* IBS1}{m}$, where IBS1 and IBS2 are the number of loci which share either 1 or 2 alleles identical by state (IBS), respectively, and *m* is the number of loci tested. Genetic distance between all pair-wise combinations of individuals was calculated as: D = 1 - D_ST_.

#### Inbreeding coefficients

The following measures of inbreeding were calculated for each breed group:

1. **Based on excess of homozygosity (F**_**EH**_ **):** $\frac{1}{m}\sum_{i=1}^m1-\frac{c_i\;\left(2-{c}_i\right)}{2{p}_i\left(1-{p}_i\right)}$, where *m* is the number of SNPs, *p*_*i*_ is the frequency of the first allele and *c* is genotype call (i.e. the number of copies of the first allele) [25].
2. **VanRaden (F**_**VR**_ **):** The F_VR_ estimate was calculated following VanRaden [26] based on the variance of additive genotypes. F_VR_ was derived from ${F}_{VR}=\frac{\sum_{i=1}^m{\left[{c}_i- E\left({c}_i\right)\right]}^2}{2{\sum}_{i=1}^m{p}_i\left(1-{p}_i\right)}-1=\frac{\sum_{i=1}^m{\left({c}_i-2{\widehat{p}}_i\right)}^2}{2{\sum}_{i=1}^m{p}_i\left(1-{p}_i\right)}-1$. This was equivalent to estimating an individual’s relationship to itself (diagonal of the SNP-derived GRM) [27].
3. **Leutenneger (F**_**LEUT**_ **):** The inbreeding coefficient for an individual is estimated as: $\frac{1}{m}\sum_{i=1}^m\;\frac{{\left({c}_i-2{p}_i\right)}^2}{2{p}_i\left(1-{p}_i\right)}$ [28].
4. **Runs of homozygosity – ROH (F**_**ROH**_ **):** ROH is also associated with inbreeding. Therefore, F_ROH_ was estimated for each individual by the sum of regions of the genome that consists of runs of homozygosity (see next section for description of ROH calculation) divided by the total genome length across all 29 autosomes [29] covered by SNP in the Goat 50K SNP chip.
5. **Pedigree-based inbreeding (F**_**PED**_ **):** The pedigrees of animals were traced back to the founder populations and mean inbreeding coefficients per breed were calculated using the Colleau’s indirect method [30].

### Identifying runs of homozygosity

Runs of homozygosity were identified and characterized using PLINK [25]. To minimize the number of ROH that could occur by chance in the 50K SNP chip, the minimum number of SNPs that constituted a ROH (*l*) was calculated following Lencz et al., [31]: $l=\frac{log_e\frac{\alpha}{n_{s.}{n}_i}}{log_e\left(1- het\right)}$, where *n*_*s*_ is the number of SNPs per individual, *n*_*i*_ is the number of individuals, *α* is the percentage of false positive ROH (set to 0.05 in the present study), *het* is the mean heterozygosity across all SNPs.

### Determination of genomic regions under selection

Combining alternative approaches to detect selection signatures has been suggested as a way of increasing the reliability of selection signature studies [32]. Therefore, we implemented F_ST_ and hapFLK statistics.

#### Single SNP and smoothed F_ST_ statistics

F_ST_ indicates a difference among groups of individuals (i.e. populations, individual breeds, breeds selected for divergent purposes) in a segment of the genome that could be caused by different selection histories. To identify population-specific loci under positive selection in goats, we calculated the F_ST_ value for each of the 48,417 informative SNPs along the genome using different contrasting groups to estimate the allelic frequencies of each group. Subsequently, the allelic frequencies were used to calculate F_ST_ as a measure of group differentiation per loci following the pipeline proposed by Porto-Neto et al. [33, 34]. In brief, for each SNP in a population, F_ST_ was calculated as the squared deviation of the average frequency in that population from the average frequency across all populations divided by the allele frequency variance (p*q).

In order to identify genome regions putatively under selection, the analyses were performed under three scenarios of contrasting models:

1. **Individual populations (F**_**ST1**_ **):** Each breed (*n* = 9) was compared against all others before the pairwise population values were averaged to obtain a single F_ST_ value per SNP for breed. The breeds were: Alpine, LaMancha, Saanen, Toggenburg, Australian Boer, Canadian Boer, Rangeland, Nubian and Cashmere.
2. **Selected breeds based on breeding goals (F**_**ST2**_ **):** Only the breeds that have undergone a more intense selection pressure for some traits were grouped together (*n* = 3). The groups were defined as: milk (Alpine and Saanen), meat (Canadian and Australia Boer populations) and fiber (Cashmere).
3. **Groups based on breeding objectives (F**_**ST3**_ **):** The groups (*n* = 4) were created based on the selection purposes and including all breeds: milk (Alpine, LaMancha, Saanen and Toggenburg), meat (Australian and Canadian Boer and Rangeland), dual purpose (Nubian) and fiber (Cashmere).

Smoothing, where a moving average is taken of a certain number of markers, is an approximate method of looking for regions where selection is apparent over multiple markers, rather than one-off high values. Individual SNP F_ST_ as calculated previously may not clearly show a strong signal. To facilitate the identification of genomic regions containing more extreme F_ST_ values, the individual SNP values of F_ST_ were then grouped within genomic windows, using a kernel regression smoothing algorithm [35] implemented in the *Lokern* package in R [36]. This method uses a local averaging of the observations (F_ST_) when estimating the regression function.

By testing windows of two, five and 10 SNPs, we chose a window of five SNPs (two on each side) as it gave sufficient smoothing and showed the best signals. Higher scores of F_ST_ for individual locus or genomic regions (smoothed F_ST_) indicates stronger signal of differentiation or selection. For each breed group within each scenario, smoothed F_ST_ values greater than the average plus three standard deviations were considered to be under selection. However, F_ST_ values greater than the average plus two standard deviations were also presented as potential regions under selection. It was also recorded whether a region was exclusive to a group or shared with others.

#### HapFLK statistics

The hapFLK approach can be applied to un-phased genotypic data. The software for calculation of distance matrices and the estimation of hapFLK is available at https://forge-dga.jouy.inra.fr/projects/hapflk and described in details by its creators [20]. A Reynolds distance matrix was calculated in order to estimate the hierarchical population structure within each population set. In this study no outgroups were defined. We prompted hapFLK software to use all populations and the midpoint as outgroup.

Reynolds distances were converted into a kinship matrix using an R script supplied with the hapFLK package. The hapFLK program was then run using the genotypes and kinship matrix assuming 10 clusters in the fastPHASE model (−k 10), before the hapFLK statistic was computed as the average across 20 expectation-maximization (EM) runs to fit the LD model (−−nfit = 20). Instead of correcting for multiple testing, an approach similar to Kijas [37] was applied. P-values were computed from the null distribution of empirical values as follows. First, the mean and variance of the hapFLK distribution was estimated and used to standardize each SNP-specific value. The distribution of the standardized hapFLK values appears to approximately fit a normal distribution (Additional file 1). P-values were computed from a standard normal distribution, and the negative log of P-values was plotted against the genomic position.

### Genomic population trees

The neighbour-joining algorithm was used to plot genomic population trees using pair-wise population Reynolds distance. Genomic population trees were created using all genome-wide SNPs (genome tree). We also created genomic population trees using only those SNPs located within the regions of signatures of selection (“local trees”) identified using the hapFLK methodology to show the breeds undergoing selection. The analysis was done following Kijas [37].

### Gene content of regions identified as under selection

The significant genomic regions revealed by smoothed F_ST_ or hapFLK were identified and lists of genes partially or fully covered by these regions were then established. Genes located in the significant genomic regions were identified using the goat reference genome assembly v1.0 (http://www.ncbi.nlm.nih.gov/genome?term=capra%20hircus).

Gene annotation was performed using Ensembl Comparative Genomics Resources (Database release 84) and NCBI Gene database. Due to the incomplete annotation of the goat genome, BioMart tool of Ensembl (www.ensembl.org/biomart) was used to determine the orthologous bovine (*Bos Taurus*), ovine (*Ovis aries*), swine (*Sus scroffa*) and human (*Homo sapiens*) gene IDs for each gene detected. The biological functions and pathways in which these genes are involved were assessed using Panther [38]. The next step was a search in the literature and in the Bovine, Pigs and Ovine QTL database available online at http://www.animalgenome.org/cgi-bin/QTLdb/index to identify phenotypes known to be affected by variation in the genes located in the peaks of each significant genomic region.

### Genome-wide association study (GWAS) for ear type

**Fig. 1:**
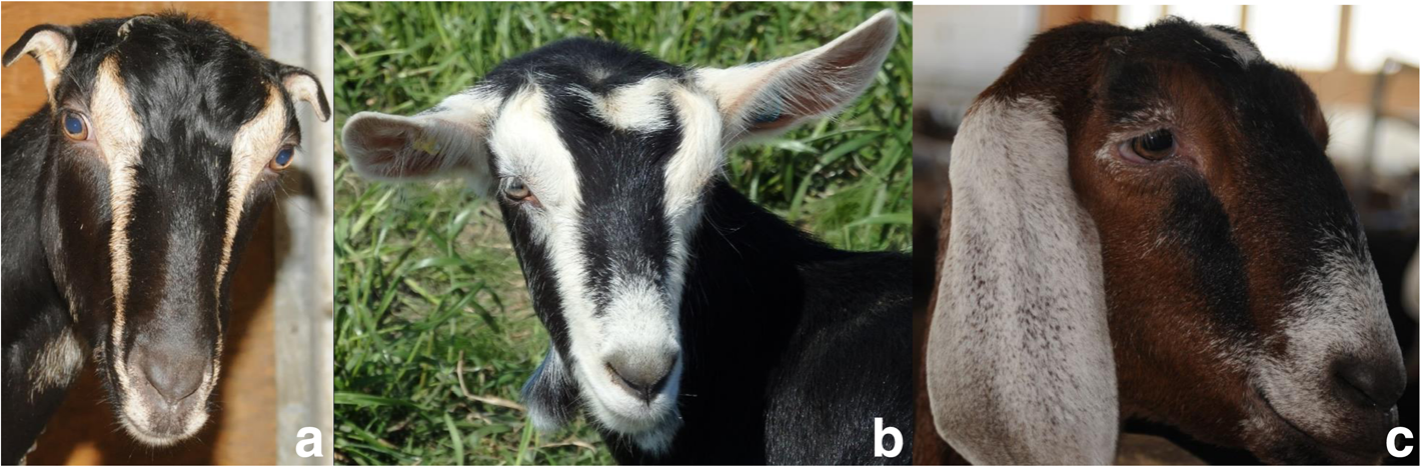
Goat breeds with different ears size. **a** LaMancha breed (*short ears*), **b** Toggenbourg breed (*average ears*) and **c** Nubian breed (*long ears*). Photo credits: Ontario Goat (http://ontariogoat.ca/)

The breed LaMancha has been intensively selected for short ears and Nubian for long and pendulous ears. We used these breed level phenotypic differences to conduct a GWAS for ear type. The phenotype for animals with short ears (LaMancha breed, Fig. 1a), average size ears (Boer, Alpine, Saanen, Cashmere, Rangeland and Toggenbourg, Fig. 1b), long ears (Nubian, Fig. 1c) was coded as 0, 1 and 2, respectively. The GWAS was conducted using a single SNP regression, including a polygenic term by fitting the genomic relationship matrix. Analysis were performed using snp1101 software [39].

## Results

### Genetic diversity metrics

Genetic diversity metrics within each of the nine populations were assessed by estimating the percentage of polymorphic SNPs, observed and expected heterozygosity, average pairwise genetic distance and genomic and pedigree inbreeding as showed in Table 1. The number of samples ranged from 48 (Cashmere) to 403 (Alpine) and included breeds selected for different purposes (i.e. meat, milk, dual-purpose, and fiber) and sampled in different geographic regions (i.e. Australia and Canada). The proportion of polymorphic SNPs ranged from 0.902 (Nubian) to 0.995 (Rangeland). The overall mean H_O_ and H_E_ was 0.374 ± 0.021 and 0.369 ± 0.023, respectively. The average H_O_ was lowest in Nubian (0.338) and highest in Rangeland (0.413). The average pairwise genetic distance (D) was used as a measure of homogeneity of samples within each breed/population, where higher values indicates a greater genetic variation within breed. D ranged from 0.263 (Toggenburg) to 0.323 (Rangeland). A summary of genetic diversity metrics using a balanced sample size (*n* = 48) is presented in Additional file 2. When using a reduced number of samples P_N_ was slightly greater. However, the changes in the other genetic diversity measures were small and therefore, we decided to present the results of the analysis including all the genotyped animals.

We used five different approaches to estimate inbreeding coefficients using information from two different sources: pedigree and 50K SNP chip genotype data. The average inbreeding coefficients estimated using different approaches and different data sets are shown in Table 1. The overall average (± SD) for F_EH_, F_VR_, F_LEUT_, F_ROH_ and F_PED_ was 0.129 ± 0.048, −0.012 ± 0.016, −0.010 ± 0.014, 0.038 ± 0.015 and 0.030 ± 0.018, respectively. The average inbreeding coefficients differed among breeds (Table 1). The Australian animals did not have pedigree recorded and therefore F_PED_ was not calculated. Levels of inbreeding for Australian Boer goats were slightly lower compared to Canadian Boer animals.

The lowest inbreeding averages for all breeds were F_VR_ and F_LEUT_, which are dependent on allele frequencies. Additional file 3 presents the allele frequency distribution for each breed. As expected, Rangeland was the breed with the lowest proportion of SNPs with low MAF and highest proportion of SNPs with high allele frequency, highlighting its genetic diversity. There was some variation among the other breeds, however, not as evident as the one observed for the Rangeland population.

Table 2 presents the Pearson correlations among the different measures of inbreeding coefficients. F_LEUT_ and F_VR_ were highly correlated (0.969). F_EH_ was also highly correlated with F_ROH_ (0.901). F_VR_ and F_LEUT_ presented a low and/or negative correlation (range: −0.264 to 0.067) with the other inbreeding measures. F_PED_ presented the highest correlation (0.372) with F_ROH_ (0.473) method and the lowest (−0.011) with F_LEUT_. The lowest correlation among all inbreeding measure pairs was between F_LEUT_ and F_EH_ (−0.318).

**Table 2:** Pearson correlations among alternative inbreeding coefficients

	F_VR_	F_LEUT_	F_ROH_	F_PED_
F_EH_	−0.132	−0.318	0.901	0.320
F_VR_		0.969	−0.133	0.067
F_LEUT_			−0.264	−0.011
F_ROH_				0.372

*F*_*EH*_ *, F*_*VR*_ *, F*_*LEUT*_ *, F*_*ROH*_ *and F*_*PED*_ inbreeding coefficients based on excess of homozygosity, VanRaden, Leutenegger, runs of homozygosity and pedigree, respectively

### Description of runs of homozygosity

Table 3 presents a descriptive summary of ROH which were observed across all 29 autosomes. The average number of ROH segments for each animal within breed ranged from 5.19 ± 3.36 (Rangeland) to 31.52 ± 7.85 (Canadian Boer), with a maximum of 59 segments in a Canadian Boer animal, followed by Nubian (46). Nubian also presented a high average of ROH segments (31.20 ± 7.20). For Cashmere and Rangeland, the maximum number of ROH segments was 16 and 19, respectively. The average length of genome contained within ROH segments ranged from 22,800 kb ± 22,370 kb (Rangeland) to 138,100 kb ± 45,131 kb (Nubian). The animal with the longest proportion of its genome characterized as ROH was observed in the Nubian breed (332,000 kb).

**Table 3:** Descriptive analysis of the runs of homozygosity per breed and including all genotyped animals

		Breed								
		AL	BA	BC	CA	LA	NU	RA	SA	TO
NSEG	Mean	15.6	23.6	31.5	8.1	19.4	31.2	5.2	16.7	24.1
	SD	8.5	7.2	7.9	2.9	7.1	7.2	3.4	8.4	9.1
	Min	0	9	0	4	6	13	0	0	2
	Max	44	45	59	16	38	46	19	43	40
KB	Mean	74,510	113,200	137,900	49,630	93,710	138,100	22,800	79,720	111,700
	SD	47,837	35,307	39,826	22,197	43,500	45,131	22,370	46,714	43,759
	Min	0	47,010	0	12,780	21,670	49,430	0	0	5731
	Max	307,100	192,100	287,600	98,230	231,900	332,000	160,400	242,500	179,200
KB_AVER_	Mean	4503	4824	4298	5967	4719	4375	3859	4518	4570
	SD	1187	740	677	1423	767	609	1933	1052	686
	Min	0	3618	0	2766	3096	3327	0	0	2866
	Max	7869	6647	5338	8595	6872	7217	12,130	8916	6108
NSNP	Mean	100.2	100.2	91.9	126.7	100.8	92.9	91.4	100.2	97.2
	SD	62.5	66.2	52.8	87.3	63.6	53.6	72.3	63.2	61.1
	Min	42	46	45	47	48	45	47	43	47
	Max	800	655	614	640	669	691	701	826	573
Density	Mean	47.5	47.5	47.3	47.7	47.5	47.3	47.6	47.4	47.3
	SD	2.1	2.3	2.3	1.9	2.1	2.3	1.9	2.0	2.2
	Min	29.8	23.5	21.5	35.6	26.8	27.9	40.5	28.5	26.7
	Max	50	50	50	50	49	50	49	50	50
PHOM	Mean	0.96	0.99	0.96	0.99	0.96	0.96	0.98	0.96	0.96
	SD	0.01	0.01	0.01	0.01	0.01	0.01	0.01	0.01	0.01

*NSEG* average number of segments for the individual declared homozygous, *KB* average of total number of kb contained within homozygous segments, *KB*_*AVER*_ average size of homozygous segments, *NSNP* average number of SNPs in run, *PHOM* proportion of sites homozygous, *AL* Alpine, *BA* Australian Boer, *BC* Canadian Boer, *CA* Cashmere, *LA* LaMancha, *NU* Nubian, *RA* Rangeland, *SA* Saanen, *TO* Toggenburg, *Min* minimum, *Max* maximum, *SD* standard deviation

The average size of homozygous segments ranged from 3859 kb ± 1933 kb (Rangeland) to 5967 kb ± 1423 kb (Cashmere). The longest segment of ROH was observed in the Rangeland breed which has high genetic diversity. It could potentially be due to recent selection or inbreeding. The average number of SNPs in run per breed ranged from 91.36 ± 72.25 (Rangeland) to 100.80 ± 63.64 (LaMancha), presenting a minimum and a maximum of 42 and 826 SNPs, respectively. The average SNP density (SNPs per kb) was similar for all breed groups (47 SNPs/kb) and the proportion of homozygous sites was higher than 96% for all breed groups.

**Fig. 2:**
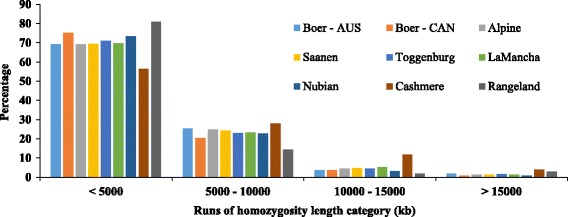
Proportion of runs of homozygosity segments in each length category for the nine goat populations. AUS: Australia; CAN: Canada

Figure 2 shows the proportion of ROH in each length category for the nine goat populations. Rangeland was the population with the higher proportion of short ROH (<5000 kb), followed by Canadian Boer and Nubian. The population with the lowest proportion was Cashmere. Alpine and Saanen presented very similar values in all categories. Cashmere and Rangeland were the breeds with the highest and lowest proportion of ROH between 5000 and 15,000 kb, respectively. However, both Cashmere and Rangeland were the populations with the highest proportion of long segments of ROH (>15,000 kb).

### Signatures of selection

**Fig. 3:**
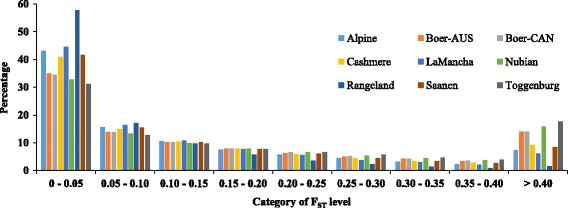
Distribution of SNP F_ST_ values within each of the nine goat populations. AUS: Australia; CAN: Canada

**Fig. 4:**
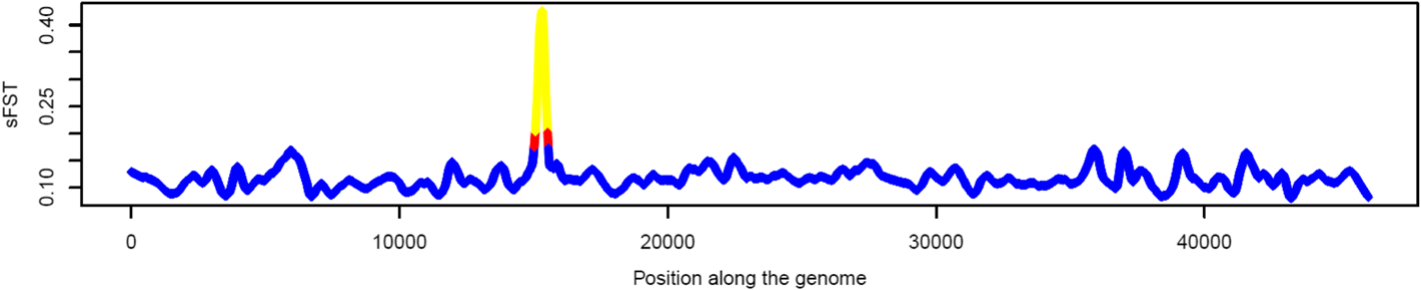
Whole genome scans for selection in the LaMancha breed compared with all other breeds using the smoothed F_ST_ approach. Smoothed F_ST_ values greater than average plus two or three standard deviations were coloured with *red* and *yellow*, respectively. Plots for the other breeds are presented in the Supplementary material section

High F_ST_ values indicate potential positive selection while low F_ST_ values suggest negative or neutral selection. There were several regions across the genome that were potentially under selection in at least one of the goat breeds. Considering F_ST_ values, these were distributed on all chromosomes, with the number of significant SNPs per chromosome varying from 110 (CHI29) to 439 (CHI7). Figure 3 present the distribution of SNP F_ST_ within each of the nine goat populations. Rangeland and Toggenburg presented the highest and lowest percentage of SNPs with F_ST_ values within the category 0 to 0.05, respectively. On the other side, a reverse trend was observed in the category “>0.40” (i.e. Rangeland presented the lowest and Toggenburg presented the highest F_ST_ values). Canadian and Australian Boer presented similar values. Alpine and Saanen breeds also had similar estimates. As previously mentioned, smoothed F_ST_ values give more accurate indication of regions under selection. Therefore, we did not present in this paper plots for the single SNP F_ST_ values. As an example of the smoothing process, Fig. 4 and Additional file 4 present single SNPs F_ST_ and smoothed F_ST_ for the LaMancha breed. Additional file 5 presents the results for all the other breeds and scenarios.

Significant peak regions were detected on 19 chromosomes through smoothed F_ST_ statistics (considering two or three standard deviations (SD) as the significance thresholds) were presented in the Tables 4, 5 and 6 and in the Additional file 6. For the scenario 1 (F_ST1_) that is designed to detect population specific sweep regions in each breed group (*n* = 9), 34 unique peaks were identified and 10 of them under a three SD threshold. Twenty seven predicted putative signatures were breed-specific and seven peaks were shared between breeds (Tables 4, 5 and 6). Common signatures of selection overlapped but did not have identical boundaries in all breeds. Australian and Canadian Boer shared only one peak (located on CHI3). Saanen, Nubian, Canadian Boer and Rangeland shared a peak on CHI6, which was highly significant (> mean + 3 SD) for Saanen and Rangeland.

**Table 4:**
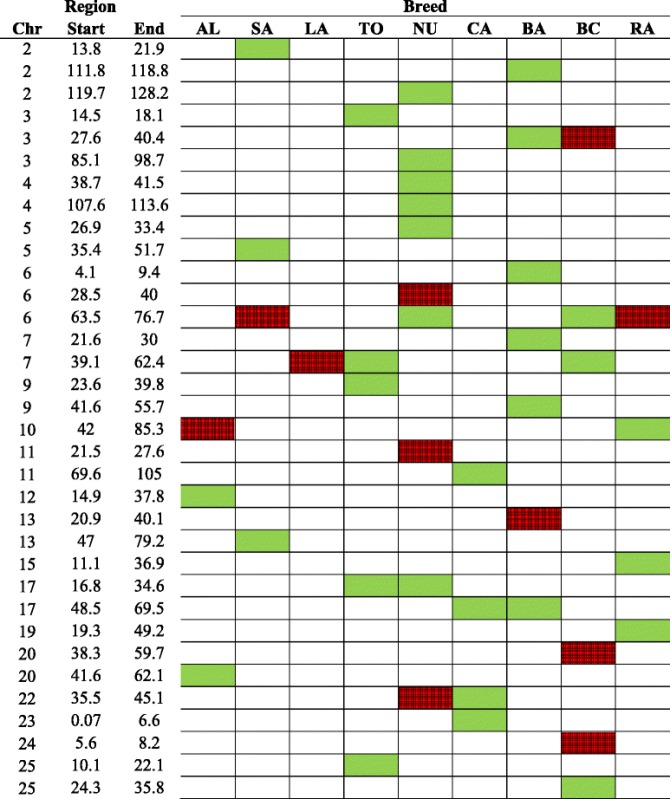
Signatures of selection for the scenario 1 (F_ST1_, all breed comparisons) identified using smoothed F_ST_ statistics and considering two (green) or three (red) standard deviations as significance threshold

*Chr* chromosome, *AL* Alpine, *BA* Australian Boer, *BC* Canadian Boer, *CA* Cashmere, *LA* LaMancha, *NU* Nubian, *RA* Rangeland, *SA* Saanen, *TO* Toggenburg

**Table 5:**
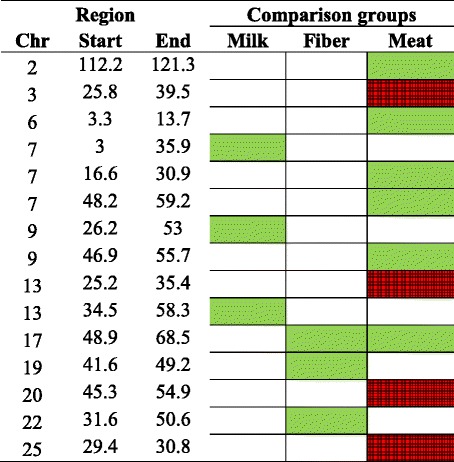
Signatures of selection for the scenario 2 (F_ST2_, selected breeds based on selection purpose) identified using smoothed F_ST_ statistics and considering two (green) or three (red) standard deviations as significance threshold

*Milk* Alpine and Saanen breeds, *Fiber* Cashmere breed, *Meat* Australian and Canadian Boer breed, *Chr* chromosome

**Table 6:**
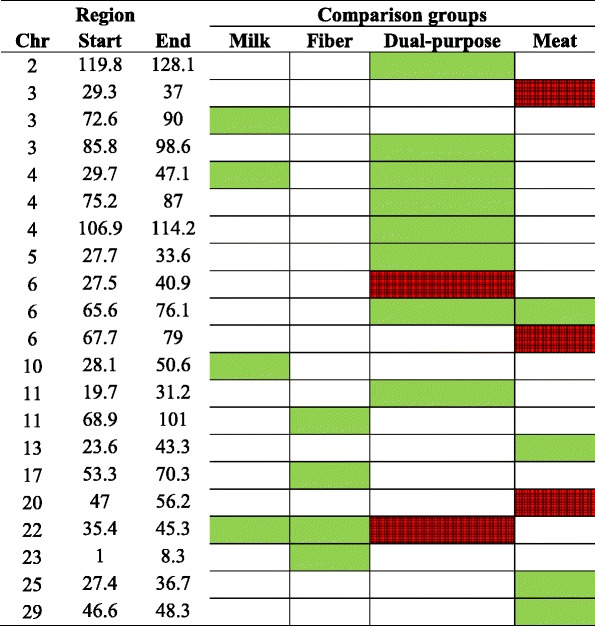
Signatures of selection for the scenario 3 (F_ST3_, breed groups based on selection purpose) identified using smoothed F_ST_ statistics and considering two (green) or three (red) standard deviations as significance threshold

*Milk* Alpine, Saanen, LaMancha and Toggenburg breeds, *Fiber* Cashmere breed, *Dual-purpose* Nubian breed, *Meat* Canadian and Australian Boer and Rangeland breeds, *Chr* chromosome

The number of selection signatures that are shared between breed groups selected for different breeding objectives could provide new insights into the discussion about the evolution of goat breeds. In the scenario 2 (F_ST2_), where we contrasted breeds under more intensive selection for milk (Alpine and Saanen), fiber (Cashmere) and meat (Australian and Canadian Boer), we observed 15 significant peaks and four of them (all in Boer animals) were highly significant (> mean + 3 SD). Only one peak on CHI17 was shared between fiber and meat breeds. For the scenario 3 (F_ST3_), where all breed groups were assigned to one selection purpose for contrasting: milk (Alpine, Saanen, LaMancha and Toggenburg), fiber (Cashmere), dual-purpose (Nubian) and meat (Australian and Canadian Boer and Rangeland), 21 significant peaks were identified and 5 of them were highly significant (> mean + 3 SD). A peak on CHI6 was shared between dual purpose and meat breeds and a peak on CHI22 was shared among milk, fiber and dual-purpose breeds. Fourteen and 16 out of the 34 peaks identified in F_ST1_, were also identified in F_ST2_ and F_ST3_, respectively. When comparing F_ST2_ and F_ST3_, 7 peaks were identified in both cases. However, F_ST3_ also included Nubian, which presented 10 significant peaks.

**Fig. 5:**
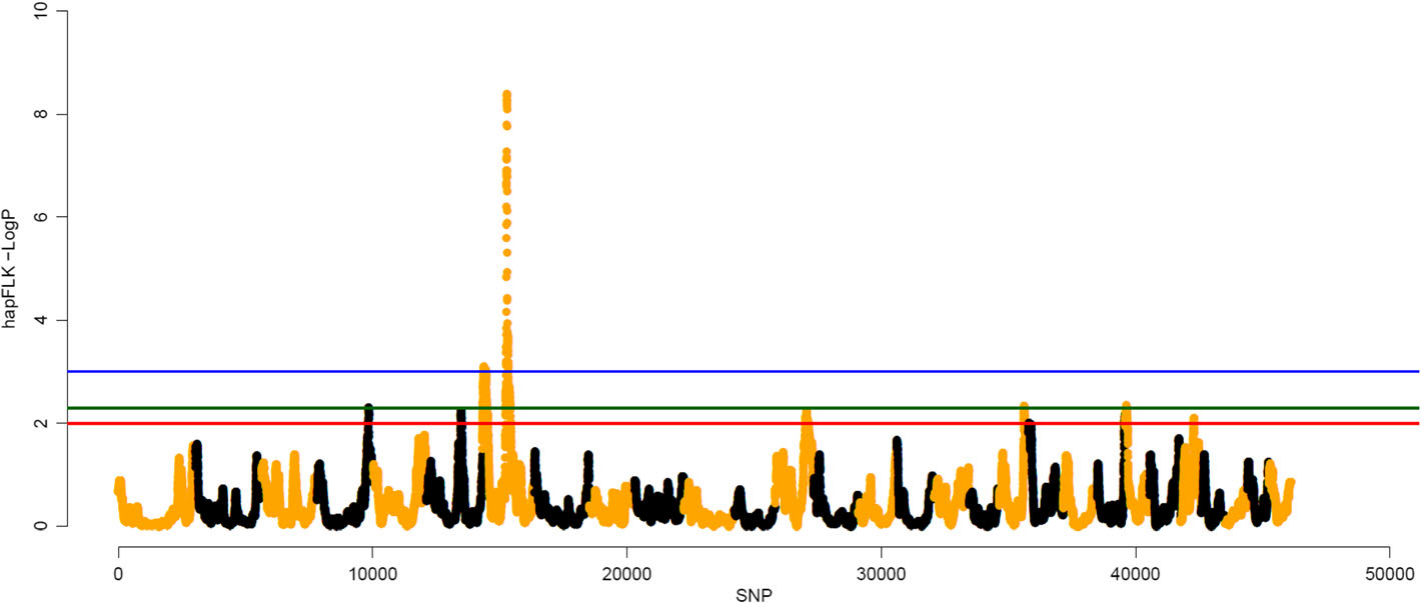
Whole genome scans for selection using the haplotype based hapFLK metric and –log (*P*-values) were plotted in genomic order. Odd and even numbered chromosomes are shown in *yellow* and *black*, respectively. SNP number is given on the x axis, and the genome-wide threshold corresponding to *P* < 0.001, *P* < 0.005 and *P* < 0.01 is shown as horizontal blue, green and red lines, respectively

Figure 5 shows the significant peaks (*p* < 0.001, *p* < 0.005 and *p* < 0.01) identified using the hapFLK metric for assessing haplotype differentiation between populations. We considered as significant peaks with *p*-values < 0.005. These peaks were located on CHI4 (105.2 to 105.7 Mb), CHI6 (73.1 to 74.0 Mb), CHI7 (0.8 to 9.4 Mb), CHI7 (48.5 to 57.3 Mb), CHI13 (66.0 to 67.2), CHI19 (54.1 to 54.5) and CHI23 (3.3 to 4.1). Additional file 7 shows the peaks identified on CHI7 in more details. Five out of seven peaks (CHI6, both on CHI7, CHI13 and CHI23) were also identified by the smoothed F_ST_ approach.

### Genomic population trees

**Fig. 6:**
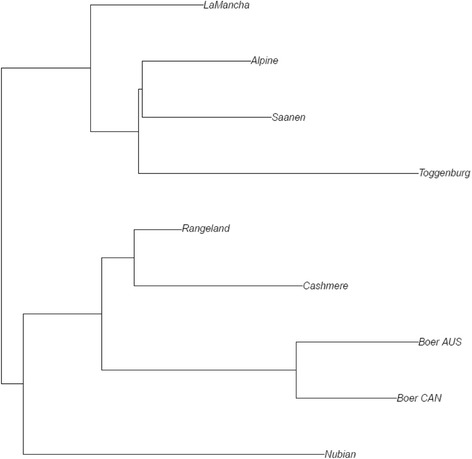
Genomic population tree using all SNPs that passed genotype quality control. AUS: Australia and CAN: Canada

**Fig. 7:**
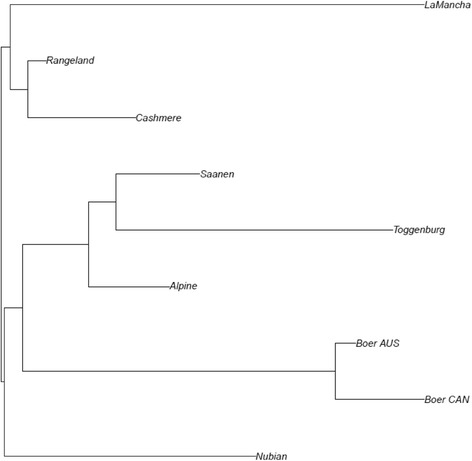
Genomic population tree using significant SNPs the most significant region on chromosome 7 (CHI7:48.4–57.3 Mb). AUS: Australia and CAN: Canada

Figure 6 shows the genomic population tree constructed using all SNPs. The top branch separates the dairy breeds, while the middle branch indicates the meat and fiber breeds and the bottom branch the dual-purpose breed (Nubian). Another hypothesis for the breeds’ separation could be due to their geographical origins. Figure 7 presents the genomic population tree built using only SNPs presented in the region CHI7:48.4–57.3, showing a great differentiation between LaMancha and the other breeds. Additional file 8 presents the genomic population trees constructed using only the SNPs located in the other significant regions identified via hapFLK approach. The topography of these “local” trees differed significantly from the “genome” population tree.

### Mapping positively selected regions to genome annotations

We looked across the genome to identify regions showing evidence of positive selection in 9 goat populations. The genome regions with smoothed F_ST_ values greater than mean plus three SD (for F_ST1_, F_ST2_ and F_ST3_) and hapFLK p-values smaller than 0.005 (hapFLK approach) were further investigated to identify genes under positive selection. There were 10, 4, 5 and 7 regions for scenarios F_ST1_, F_ST2_, F_ST3_ and hapFLK, respectively, which were located on CHI3, CHI6, CHI7, CHI10, CHI11, CHI13, CHI20, CHI22, CHI24 and CHI25 (Tables 4, 5 and 6 and Fig. 5).

Additional file 6 shows all the genes located on each significant region. However, due to the extensive number of genes in some regions identified through smoothed F_ST_, only genes located in the regions of the top 10 most significant SNPs were shown (Tables 7, 8 and 9). The significant regions were sufficiently broad with the number of genes per region ranging from zero to 401, with an average (± SD) of 84.38 ± 102.51 genes. The average size (± SD) of the regions was 9.9 ± 9.1 Mb. Some of the genes located in the peak regions have been reported as potentially associated with important traits. For instance, genes related to fertility and reproduction traits (e.g. *CACNB2* [40], *MEF2BNB* [41] and *CYP19A1* [42–45]), adult body mass (e.g. *GPR61* [46]), post-weaning gain (e.g. *MEF2B* [47]), efficiency of food conversion (e.g. *KIAA1211* [48] and *VAV3* [49]), abdominal fat deposition (e.g. *PRPSAP1* [50]), conformation traits (e.g. *RNF157* [51]), liver fat metabolism (e.g. *TM6SF2* [52]), mineral concentration in muscle tissue (e.g. *TRNAC-GCA* [53]), milk fatty acids (e.g. *CDH12* [54]), somatic cells score and milk protein (e.g. *FAM13A* [55, 56]), thermo-tolerance (e.g. *GNAI3* [57]) and longissimus muscle development in bovine fetuses (e.g. COL12A1, [58]). Other interesting genes were also present in the signature of selection sweeps such as *SIX2*, which has been associated with outer ear development [59–64] and *WNT5A* which has been associated with ear morphogenesis [65].

**Table 7:** Genomic regions under differential selection for all goat breed comparisons (F_ST1_) and list of the genes located in the region of the 10 most significant SNPs (based on smoothed F_ST_ values)

Scenario	Breed	Chr	Size (Mb)	Ngenes	Peak SNP	Genes present in the region of the 10 peak SNPs
F_ST1_	AL	10	43.4	401	snp25170-scaffold259-4130319	*CYP19A1*, *LOC102172726*, *TNFAIP8L3*, *AP4E1*
F_ST1_	BA	13	19.2	137	snp49043-scaffold7-3464730	*ITGA8*, *FAM188A*
F_ST1_	BC	3	12.9	133	snp46905-scaffold654-1593334	*LOC102168226*
F_ST1_	BC	20	21.4	63	snp34011-scaffold40-2937263	*CDH12*
F_ST1_	BC	24	2.6	7	snp836-scaffold1022-339033	No genes in the peak region
F_ST1_	LA	7	23.3	353	snp23253-scaffold2322-180232	*KCTD16*
F_ST1_	NU	6	11.4	53	snp16822-scaffold1760-904920	*FAM13A*, *HERC3*, *NAP1L5*
F_ST1_	NU	11	6.2	37	snp9748-scaffold135-1883590	*KCNG3*, *MTA3*, *LOC102176890*, *OXER1*, *HAAO*, *TRNAI-UAU*
F_ST1_	NU	22	9.6	47	snp15314-scaffold163-1176183	*FHIT*
F_ST1_	RL	6	3.6	7	snp11723-scaffold1432-75297	*LOC102184415*, *TRNAC-GCA*
F_ST1_	SA	6	13.2	86	snp58130-scaffold94-7008249	*CEP135*, *KIAA1211*, *AASDH*, *PPAT*, *LOC102176867*, *PAICS*, *SRP72*, *LOC102177146, ARL9, THEGL, LOC102178156, LOC102178436, HOPX*

*AL* Alpine, *BA* Australian Boer, *BC* Canadian Boer, *CA* Cashmere, *LA* LaMancha, *NU* Nubian, *RA* Rangeland, *SA* Saanen, *TO* Toggenburg, *Chr* chromosome, *Ngenes* total number of genes in the whole significant region

**Table 8:** Genomic regions under differential selection based on breeds grouped per selection purpose and list of the genes located in the region of the 10 most significant SNPs (based on smoothed F_ST_ values)

Scenario	Group	Chr	Size (Mb)	Ngenes	Peak SNP	Genes present in the region of the 10 peak SNPs
F_ST2_	Meat	3	13.7	149	snp25334-scaffold261-808539	*LOC100861222, LOC102185621, AMPD2, GNAT2, GNAI3, GPR61, AMIGO1, LOC102191753, ATXN7L2, SYPL2, LOC102169721, PSMA5, SORT1, MYBPHL, PSRC1, CELSR2*
F_ST2_	Meat	13	10.2	66	snp48991-scaffold7-1097529	*MRC1, SLC39A12, CACNB2*
F_ST2_	Meat	20	9.6	17	snp57443-scaffold916-509375	No genes
F_ST2_	Meat	25	1.4	3	snp9539-scaffold1343-1758601	*TRNAC-GCA*
F_ST3_	DP	6	13.3	60	snp16815-scaffold1760-560278	*GPRIN3, TIGD2, FAM13A, HERC3*
F_ST3_	DP	22	10.0	48	snp15310-scaffold163-1010990	*FHIT*
F_ST3_	Meat	3	7.7	96	snp46886-scaffold654-758578	*VAV3*
F_ST3_	Meat	6	11.3	52	snp2176-scaffold1066-801755	No genes
F_ST3_	Meat	20	5.4	12	snp33977-scaffold40-1436358	*LOC102188712, TRNAC-GCA, LOC102188978*

*Ngenes* total number of genes in the whole significant region, *DP* dual-purpose, *F*_*ST2*_ milk (Alpine and Saanen breeds), fiber (Cashmere breed) and meat (Australian and Canadian Boer breed), *F*_*ST3*_ milk (Alpine, Saanen, LaMancha and Toggenburg breeds), fiber (Cashmere breed), dual-purpose (Nubian breed) and meat (Canadian and Australian Boer and Rangeland breeds)

**Table 9:** Genomic regions under differential selection based on hapFLK methodology and list of the genes located in the region of the 10 most significant SNPs

Scenario	Breed	Chr	Size (Mb)	Ngenes	Peak SNP	Genes present in the region of the 10 peak SNPs
hapFLK	All breeds	4	0.5	0	snp5822-scaffold1205-140455	No genes
hapFLK	All breeds	6	0.0	0	snp2181-scaffold1066-1048134	No genes
hapFLK	All breeds	7	8.6	255	snp12700-scaffold1488-1106620	*PBX4, CILP2, NDUFA13, LOC102187668, GATAD2A, MAU2, SUGP1, TM6SF2, NCAN, NR2C2AP, RFXANK, MEF2BNB, MEF2B, TMEM161A, SLC25A42*
hapFLK	All breeds	7	8.9	92	snp57917-scaffold938-380104	*LOC102169003*
hapFLK	All breeds	13	0.0	0	snp7739-scaffold1278-1991262	No genes
hapFLK	All breeds	19	0.3	18	snp3321-scaffold1101-888266	*UBE2O, SPHK1, LOC102185500, LOC102185776, PRPSAP1, QRICH2, UBALD2, LOC102186516, RNF157, LOC102186798, FOXJ1, EXOC7, ZACN, GALR2, SRP68, EVPL, CDK3, TEN1*
hapFLK	All breeds	23	0.8	2	snp23628-scaffold2385-317663	*DST, COL21A1*

*Chr* chromosome, *Ngenes* total number of genes in the whole significant region

**Fig. 8:**
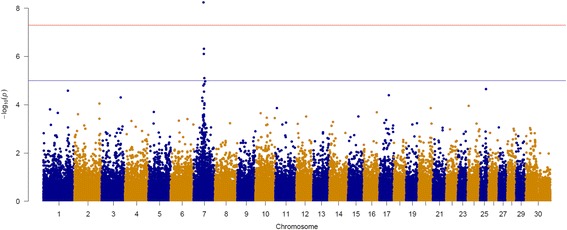
Manhattan plot for genome-wide association studies for ear size (defined as short, medium or long). *Blue and red line* indicates 0.05 and 0.01 significance threshold levels (p-values), respectively

The most significant peak was identified on chromosome 7 by both smoothed F_ST_ and hapFLK. The selection event appears to be specific for the LaMancha breed, which is a breed that has been strongly selected for short ears [66]. Therefore, we hypothesize this putative selection has acted to specifically effect ear morphology. To further explore this genome region, the levels of linkage disequilibrium (LD, r^2^) were estimated in this region for each population separately (Table 10). As expected, LaMancha had the highest LD between adjacent SNPs (0.641 ± 0.358), while the other breeds had an average of 0.246. LaMancha had a level of syntenic SNPs LD in this region of 0.263, while the other breeds presented an average of 0.095, within the haplotype block, consistent with selection being imposed on the locus. Table 10 also shows the number of SNPs in the region after the QC, which ranged from 112 for LaMancha to 187 for Rangeland. This is another indication of a higher proportion of alleles with very low MAF (<0.05) in this region in the LaMancha breed. As a complementary analysis, GWAS for ear type was performed. Figure 8 shows the Manhattan plot for GWAS for ear size (short, medium or long). After a 1 and 5% genome-wise False Discovery Rate adjustment there were 1 (snp10026-scaffold1356-1762329) and 3 (snp10026-scaffold1356-1762329, snp57913-scaffold938-217487 and snp9990-scaffold1356-259196) significant SNPs on CHI7, respectively. Positional candidate genes located in the region that support our hypothesis are: *CXCL14* (ear development), *POU4F3* (ear morphogenesis), *NDST1* (organ morphogenesis), *PPP2CA* (mesoderm development), *PITX1* and *SMAD5* (cartilage development), *ANXA6* (growth plate cartilage chondrocyte differentiation) and *HAND1* (cartilage morphogenesis) gene.

**Table 10:** Linkage disequilibrium (r^2^) levels for all the breeds in the peak region of chromosome 7

		Average linkage disequilibrium (r^2^)	
Breed	NSNP after QC	Adjacent SNPs (± SD)	Syntenic SNPs (± SD)
AL	179	0.169 ± 0.218	0.054 ± 0.077
BA	173	0.364 ± 0.322	0.134 ± 0.179
BC	164	0.392 ± 0.316	0.154 ± 0.193
CA	178	0.171 ± 0.212	0.085 ± 0.120
LA	112	0.641 ± 0.358	0.263 ± 0.289
NU	154	0.253 ± 0.281	0.091 ± 0.137
RA	187	0.130 ± 0.194	0.031 ± 0.051
SA	179	0.229 ± 0.269	0.087 ± 0.119
TO	163	0.265 ± 0.289	0.126 ± 0.159

*AL* Alpine, *BA* Australian Boer, *BC* Canadian Boer, *CA* Cashmere, *LA* LaMancha, *NU* Nubian, *Pop* population, *RA* Rangeland, *SA* Saanen, *TO* Toggenburg, *QC* genotype quality control, *SD* standard deviation

Due to the large size of the significant regions Panther plots of the biological pathways represented within genes located in all the significant regions were also shown (Additional file 9).

## Discussion

### Genetic diversity metrics

A genomic characterization of genetic diversity of breeds represents a key point to design/update breeding programs and conservation strategies. We found that all breeds sustained high levels of genetic variability. Firstly, it could be due to the fact that goats have not undergone intensive selection as experienced in other species (e.g. cattle). Secondly, it could be due to the greater genetic diversity of goat breeds ancestors. The Rangeland breed was more genetically diverse than all others, which is consistent with its population history as Rangeland goats are unmanaged feral goats, which contain genetic contributions of a variety of breeds [24]. The highest levels of D observed for Rangeland indicates a higher heterogeneity within the population. On the other side, the lowest value observed for the Toggenburg breed might be explained by artificial selection, small sample size and inbreeding compared to other populations.

A high proportion of polymorphic SNPs was observed for all breeds, indicating that most SNPs are segregating in all breeds included in this study. The differences in heterozygosity levels among the breeds can be partially explained by the 50K SNP array design, which did not include all the breeds evaluated in this study and therefore, an ascertainment bias could have been added to the estimates. The panel was developed from sequence data from Saanen, Alpine, Creole, Boer, Kacang, and Savanna animals (http://www.goatgenome.org/). The genetic diversity measures observed in these nine populations are in agreement with estimates reported in the literature [15, 67, 68]. For instance, Nicoloso et al. [15] reported levels of P_N_, H_O_, H_E_ and F_EH_ ranging from 0.951 to 0.997, 0.35 to 0.41, 0.35 to 0.41 and −0.06 to 0.070, respectively, for 14 Italian goat breeds using the same SNP chip. Isolation by geographical distance can play an important role in shaping the differentiation of breeds. However, Canadian and Australia Boer still present very similar genetic diversity estimates, which is probably due to the recent isolation among the populations and probably similar population management practices.

Monitoring and controlling inbreeding is important to limit the potential impact of deleterious alleles, inbreeding depression, and loss of variance. The levels of inbreeding varied among breeds/populations and differed with methods. Overall, the levels of inbreeding are low, however, there were animals with high inbreeding coefficients. Therefore, inbreeding coefficient is a parameter that should be taken into account when planning mattings. The lowest inbreeding averages for all breeds were the inbreeding coefficients which are dependent on allele frequencies (i.e. F_VR_ and F_LEUT_). Slight negative average might be expected with genomic inbreeding when the sample size is small, which is the case for some breed groups. Another point is that for calculating genomic inbreeding we should use allele frequencies in base population [26], which is not known and because of that we simply use observed frequencies. Observed frequencies in small samples may deviate a lot from base population frequency.

The high correlation between F_LEUT_ and F_VR_ was expected as their formulae are similar. F_EH_ and F_ROH_ were also highly correlated among themselves. One justification for the negative correlation observed between F_VR_ and F_LEUT_ with the other inbreeding measures might be that some inbreeding coefficients reflect more distant inbreeding while others more recent inbreeding. Therefore, when there is more recent inbreeding in a genome there would be less distant inbreeding in that genome causing negative correlation. Recent admixture could also be another explanation for the negative correlation. Zhang et al. [27] also reported negative correlations between F_VR_ and F_EH_ of −0.83, −0.89 and −0.66 for Holstein, Jersey and Danish Red Cattle, respectively. As discussed by Zhang et al. [27] F_VR_ tends to be less accurate for populations with a low MAF and F_EH_ tends to be less accurate for populations with a high level of heterozygosity. For populations with these characteristics, a higher sample size would be needed to obtain a better estimate of inbreeding measures. When more animals from the populations under investigation are genotyped, these measures may be re-estimated and compared to the ones reported in this study.

The intermediate correlations observed between F_PED_ and F_EH_ or F_ROH_ may be partly explained by the depth of the pedigree. However, it is important to highlight that even though F_EH_ and F_ROH_ directly reflect homozygosity on the genome and is not affected by estimates of allele frequency and depth/completeness of pedigree [27], they are not true measures of inbreeding and therefore, this difference among them was expected. A similar trend was also reported by Zhang et al. [27] while studying three cattle breeds. Purfield et al. [69] also observed a positive, but higher correlation (*r* = 0.75) between the F_ROH_ and F_PED_ estimates of inbreeding in a study with cattle data. F_PED_ presented the highest correlation with F_ROH_, indicating that F_ROH_ could be a more appropriate measure of IBD alleles. In a study with pigs, Zhang et al. [70] compared five alternative estimators of individual inbreeding coefficients and observed correlations greater than 0.57 among them all. However, the authors also concluded that some measures are more relatively difficult to estimate because they require estimates of allele frequencies in the base population or a number of user-defined parameters.

ROH arise from an increased level of relatedness between individuals within a population or through positive selection [71]. Estimates of ROH can not only be used to assist with the interpretation of the inbreeding coefficient, but also to give insights about populations’ history [69, 72, 73]. As presented by Purfield et al. [69] relatively short ROH are most likely correlated to an ancestral inheritance or potential ancient bottleneck, whereas long ROH are more likely associated with relatively recent inbreeding. However, from our knowledge there are no studies evaluating ROH in goat populations. The greatest frequency in the longer ROH categories for Cashmere is an indication of more recent inbreeding. This could also be a reflection of the sampling process, where this breed had the smallest sample size and the animals sampled could be more related than the average population by chance. A higher proportion of ROH in shorter ROH categories indicates that the breeds Rangeland, Canadian Boer and Nubian could have been initially established by small founding populations but were not particularly highly affected by recent inbreeding. Selection also plays a role in the frequency of ROH in the genome. As expected, Rangeland and Cashmere, which are the breeds under less pressure of artificial selection, presented smaller number of ROH segments. Kim et al. [29] reported a significantly lower mean number of ROH per individual in an unselected Holstein population compared to two heavily selected populations in the United States, which is in agreement with our findings.

### Signatures of selection and identification of candidate genes

Using different methods we identified various regions across the genome that are potentially under selection in at least one goat breed. The reduced number of regions in some breeds could be due to a less intensive selection process or it could be due to the fact that the traits under selection could be very polygenic and therefore have not left strong signatures on their genome. It is also possible that short (and therefore old) selection sweeps are too small to be detected using the collection of around 50,000 SNPs used which are on average separated by 40–60 kb. Identifying signatures of selection for complex traits influenced by hundreds of loci under weak selection pressure can be a difficult task, as discussed in Kemper et al. [74]. Regarding to the detection methods, there were some overlapping between regions identified using smoothed F_ST_ and hapFLK approaches. As discussed by Fariello et al. [17] these tests could capture different signals. For instance, hapFLK may not capture ancient signatures of selection, for which the mutation-carrying haplotype is small and do not include many SNPs on the SNP chip panel. On the other side, single-SNP tests may fail to identify signals of selection when a single SNP is not in high LD with the causal mutation for the trait under selection. Even though there were no reports on signatures of selection in goats using hapFLK, this method has been successfully used in other studies [17, 20, 37]. The use of the F_ST_ statistic is advantageous when there is a large difference in allele frequencies across populations [75]. The higher number of significant regions observed in the Nubian breed could be due to the selection process for milk and meat production that this breed has undergone. Furthermore, in comparison to the other breeds from this study, Nubian is a more distinct breed, with long ears, higher stature and a more diverse pattern of coloration.

The environment and management conditions in which animals are raised vary among and even within countries, which could lead to higher selection pressure in different goat genomic regions in different populations. In order to verify this hypothesis, we studied Boer animals originated from Canada and Australia. Interestingly, only one region located on CHI3 overlapped between the two populations. Despite the recent divergence of the two populations, it could be an indication of selection for different traits such as tolerance to cold or warm weather. Using more breeds for each selection purpose, as done in scenarios F_ST2_ and F_ST3_, may indicate specific history of selection for each breeding goal, instead of population-specific selection histories. These scenarios could facilitate the interpretation of signatures of selection.

Both hapFLK and smoothed F_ST_ approaches have identified a highly significant peak on CHI7. In the scenario where single breeds were contrasted against each other, this high peak was observed in the LaMancha breed. The LaMancha breed has undergone an intensive process of selection for short ears. To further understand this high peak, we estimated the levels of LD in the region, which was much higher for LaMancha compared to all other breeds, indicating that there is a lower rate of recombination in this region for the LaMancha breed. Furthermore, LaMancha presented more fixed markers, which is consistent with selection signature theory, in which beneficial alleles undergoing positive selection are fixed or in the process of fixation in the population. The second step was to look for candidate genes. However, there are 353 genes located in this region, preventing us from making any conclusive assumption. The next step was to look for biological pathways in which these genes are involved. There are two interesting pathways which are biogenesis and developmental process. We believe that due to the recent selection for short ears, a long haplotype has been transmitted through generations. Over time, due to recombination events, this haplotype will be reduced to only harbor the causal mutation responsible for short ears. We also performed a GWAS for ear type and we identified 3 significant SNPs in the same region. Furthermore, when we plotted the population tree using only the significant SNPs in this region, there was a clear separation between LaMancha (short ears), other breeds with medium size ears and Nubian with long and pendulous ears. We also identified some genes that could be related to the ear morphogenesis as well. Therefore, even though we cannot be certain of this assumption, we do believe that this peak is a signature of selection left in the genome due to the selection for short ears in the LaMancha breed. Even though there are no scientific reports, it has been observed that crosses between LaMancha and other breeds also present short ears phenotype, suggesting a dominance effect of this trait. The effects of selection on the genetic variation of a specific trait can be confounded with demographic events [76]. For instance, adaptive hitchhiking, population expansion and population reduction (e.g. bottlenecks) can also result in an excess of rare alleles [77]. However, as this peak on CHI7 was identified by more than one method, the chances that this is a false positive are low.

The majority of the significant regions identified in this study had candidate genes, which indicates selection events in goats. However, most of the regions identified in this study were quite long and therefore included many genes. The threshold used in this study to determine significant regions (mean plus two or three SD) via smoothed F_ST_ has been previously recommended by Porto-Neto et al. [78]. The high number of genes identified in our study makes it difficult to comment on possible candidate genes. The currently incomplete annotation of the goat genome is another barrier for genes and/or biological pathways under selection in goats. In addition, several annotated genes were not identified (i.e., no known orthologues, gene identifier starting with “LOC”). Therefore, many genes potentially under selection could not be included in our gene ontology analyses. Despite these restrictions, sets of candidate genes were still identified in the nine goat populations under study.

The International Goat Genome Consortium (http://www.goatgenome.org/) is working towards a better annotation of the goat genome. Furthermore, as more phenotypic and genotypic data are collected around the world, we hope that our work will motivate new studies to unravel the underlying mechanisms involved in the traits under selection, as suggested by our findings. Analysis of signatures of selection are advantageous for the initial localization of genome regions, however, they have limitations for the identification of biological processes involved. Although we are limited by the ascertainment bias and low genomic coverage of our SNP dataset, we were still able to provide a list of potential genes under selection in goats, which will be the foundation for future investigations. Further investigations using larger and more complete datasets (e.g. larger number of breeds and phenotypes) are needed to confirm the role and the specific function of these highlighted candidate genes in goats selected for different breeding purposes.

## Conclusions

We presented a comprehensive description of genetic diversity measures in various worldwide common goat breeds. In general, moderate to high levels of genetic variability were observed. However, some recommendations were done regarding monitoring levels of inbreeding in breeds under more intensive selection. A characterization of runs of homozygosity also gave insights about the breeds’ history. We also identified various genome regions under positive selection using smoothed F_ST_ and hapFLK statistics and suggested genes associated with outer ear development, fertility and reproduction traits, conformation traits, efficiency of food conversion, milk fatty acids, somatic cells score and milk protein as potentially under selection. These results can now provide a foundation to formulate biological hypotheses related to selection processes in goats. Further studies are needed to confirm and refine our results by using larger populations and other technologies/methodologies such as whole genome sequencing, candidate gene sequencing, high-density SNP genotyping, gene expression profiling, and phenotypic and physiological data.

## Additional files

Additional file 1: Figure S1. Distribution of hapFLK values; **Figure S2.** Distribution of hapFLK p-values, and **Figure S3.** Distribution of standardized hapFLk values (Z-hapFLK). (DOCX 269 kb)
Additional file 2: Summary of genotyped animals and genetic diversity compared between nine goat populations and same sample size (*n* = 48) for all of them. (DOCX 13 kb)
Additional file 3: Percentage of SNP in each allele frequency category calculated individually per breed. CAN: Canadian animals; AUS: Australian animals. (DOCX 16 kb)
Additional file 4: F_ST_ (blue dots) and smoothed F_ST_ (white line) values for the LaMancha breed. (DOCX 188 kb)
Additional file 5: Smoothed F_ST_ for all the scenarios investigated and for all the breeds included in this study. (DOCX 2305 kb)
Additional file 6: Genomic regions under differential selection for various goat breeds selected for different breeding objectives. (XLSX 39 kb)
Additional file 7: Whole genome scans for selection using the haplotype based hapFLK metric and –log (*P*-values) were plotted in genomic order only for the chromosome 7 (highest peaks). SNP number is given on the x axis, and the genome-wide threshold corresponding to *P* < 0.001, *P* < 0.005 and *P* < 0.01 is shown as horizontal blue, green and red lines, respectively. (DOCX 124 kb)
Additional file 8: Population tree using significant SNPs for each signature of selection region. (DOCX 801 kb)
Additional file 9: Panther plot of the biological pathways represented within genes located in each significant region identified in this study. (DOCX 101 kb)

## Abbreviations

AL: Alpine
BA: Australian Boer
BC: Canadian Boer
CA: Cashmere
CAAP: Canadian Agricultural Adaptation Program
CCSI: Canadian Centre for Swine Improvement
D_ST_: Pairwise genetic distance
F_EH_: Inbreeding coefficients based on excess of homozygosity
F_LEUT_: Inbreeding coefficients based on Leutenneger
F_PED_: Inbreeding coefficients based on pedigree
F_ROH_: Inbreeding coefficients based on runs of homozygosity
F_VR_: Inbreeding coefficients based on VanRaden
GWAS: Genome-wide association study
H_E_: Expected heterozygosity
H_O_: Observed heterozygosity
KB: Average of total number of kb contained within homozygous segments
KB_AVER_: Average size of homozygous segments
LA: LaMancha
MAF: Minor allele frequency
Max: Maximum
Min: Minimum
MLA: Meat and Livestock Australia
NA: Not available
NSEG: Average number of segments for the individual declared homozygous
NSNP: Average number of SNPs in run
NU: Nubian
PHOM: Proportion of sites homozygous
P_N_: Proportion of polymorphic SNPs
RA: Rangeland
ROH: Runs of homozygosity
SA: Saanen
SD: Standard deviation
SNP: Single nucleotide polymorphisms
TO: Toggenburg
