# Supplementary material for: Genetic diversity and signatures of selection in various goat breeds revealed by genome-wide SNP markers
Source: BMC Genomics. 2017 Mar 14;18:229. doi: 10.1186/s12864-017-3610-0 (PMC5348779; doi:10.1186/s12864-017-3610-0)
Supplement: Additional file 1: Figure S1. — Distribution of hapFLK values; Figure S2. Distribution of hapFLK p-values, and Figure S3. Distribution of standardized hapFLk values (Z-hapFLK). (DOCX 269 kb) [file 12864_2017_3610_MOESM1_ESM.docx]

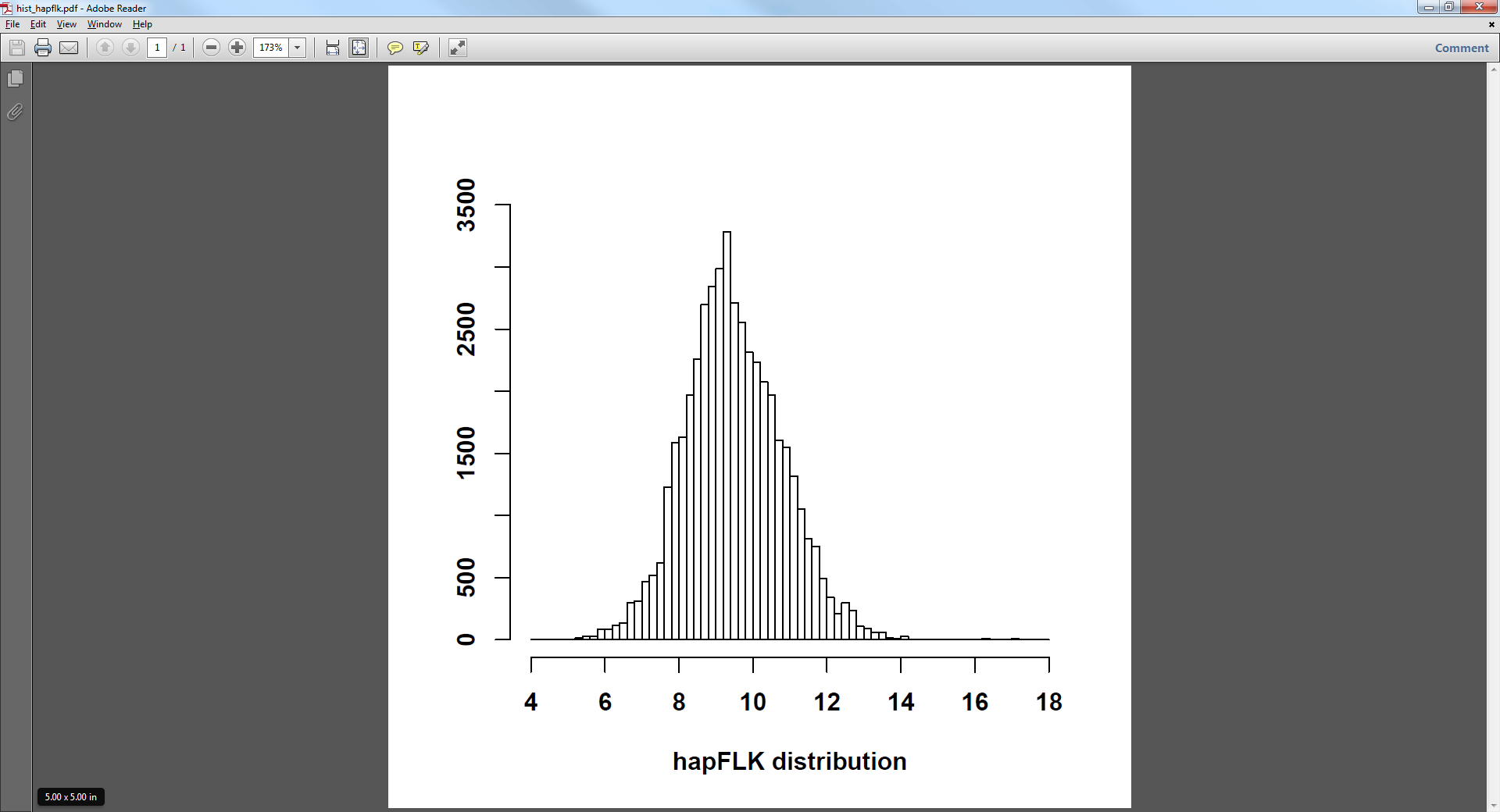


**Figure S1.** Distribution of hapFLK values.


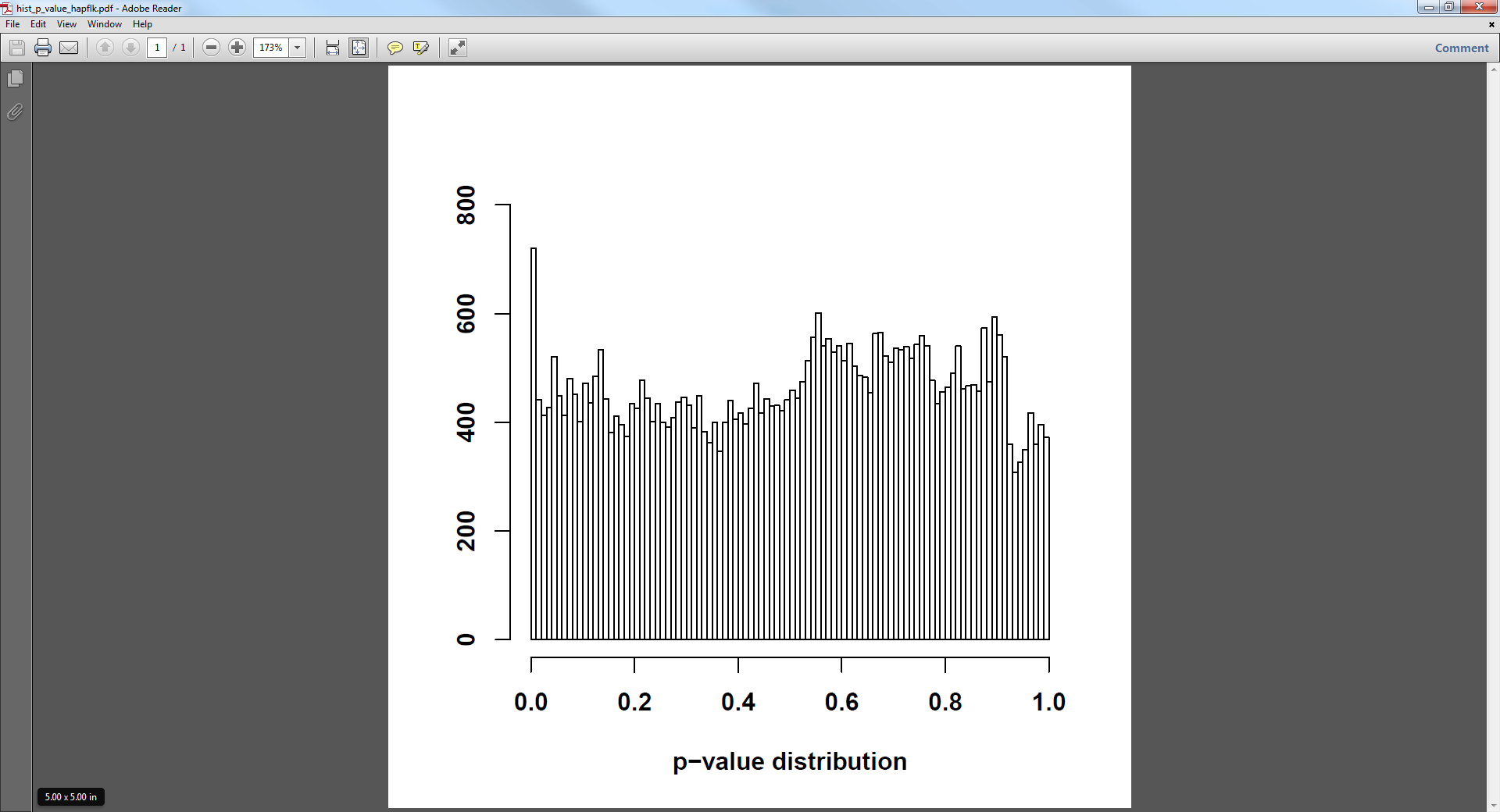


**Figure S2.** Distribution of hapFLK p-values.


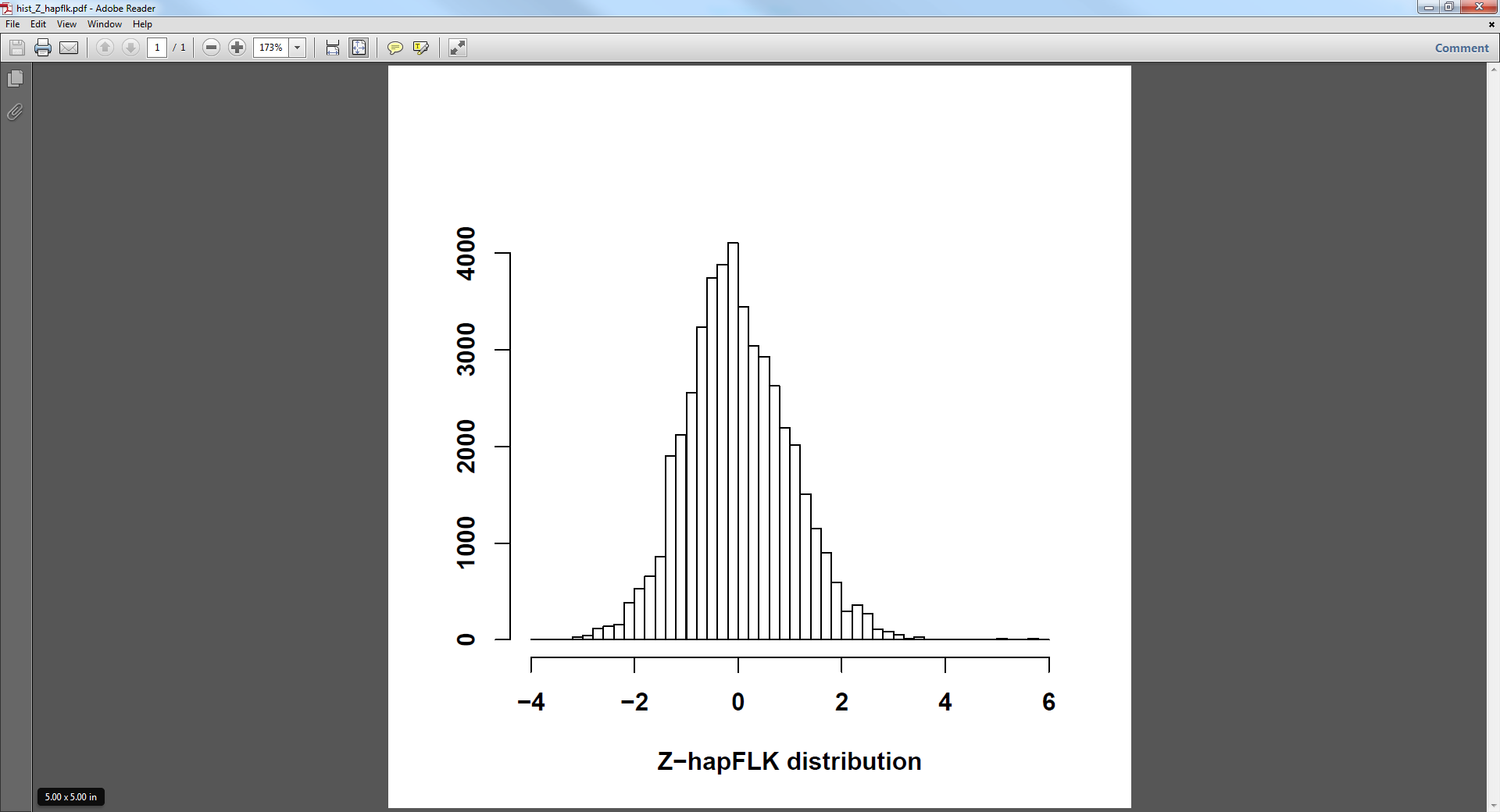


**Figure S3.** Distribution of standardized hapFLk values (Z-hapFLK).
