# Supplementary material for: Genetic diversity and signatures of selection in various goat breeds revealed by genome-wide SNP markers
Source: BMC Genomics. 2017 Mar 14;18:229. doi: 10.1186/s12864-017-3610-0 (PMC5348779; doi:10.1186/s12864-017-3610-0)
Supplement: Additional file 2: — Summary of genotyped animals and genetic diversity compared between nine goat populations and same sample size (n = 48) for all of them. (DOCX 13 kb) [file 12864_2017_3610_MOESM2_ESM.docx]

| **Table S1.** Summary of genotyped animals and genetic diversity compared between nine goat populations and same sample size (n=48) for all of them. | | | | | | | | | |
| --- | --- | --- | --- | --- | --- | --- | --- | --- | --- |
| **Breed** | **Alpine** | **Boer** | **Boer** | **Cashmere** | **LaMancha** | **Nubian** | **Rangeland** | **Saanen** | **Toggenburg** |
| **Origin** | Canada | Australia | Canada | Australia | Canada | Canada | Australia | Canada | Canada |
| **Abbreviation** | AL | BA | BC | CA | LA | NU | RA | SA | TO |
| **Sample size** | 48 | 48 | 48 | 48 | 48 | 48 | 48 | 48 | 48 |
| **P_N_** | 0.995 | 0.972 | 0.973 | 0.981 | 0.991 | 0.966 | 0.997 | 0.993 | 0.978 |
| **H_O_** | 0.381 | 0.371 | 0.361 | 0.384 | 0.386 | 0.339 | 0.416 | 0.379 | 0.353 |
| **H_E_** | 0.382 | 0.355 | 0.354 | 0.372 | 0.384 | 0.336 | 0.411 | 0.377 | 0.337 |
| **D_ST_** | 0.305 | 0.279 | 0.282 | 0.293 | 0.306 | 0.271 | 0.323 | 0.302 | 0.265 |
| **F_EH_ ± SD** | 0.12±0.06 | 0.15±0.04 | 0.16±0.04 | 0.10±0.04 | 0.11±0.05 | 0.23±0.05 | 0.04±0.05 | 0.13±0.05 | 0.19±0.06 |
| **P_N_:** proportion of polymorphic SNPs; **H_E_** and **H_O_**: expected and observed heterozygosity, respectively; **D_ST_**: average pairwise genetic distance; **F_EH_:** inbreeding coefficient based on excess of homozygosity. | | | | | | | | | |
