# Supplementary material for: Genetic diversity and signatures of selection in various goat breeds revealed by genome-wide SNP markers
Source: BMC Genomics. 2017 Mar 14;18:229. doi: 10.1186/s12864-017-3610-0 (PMC5348779; doi:10.1186/s12864-017-3610-0)
Supplement: Additional file 3: — Percentage of SNP in each allele frequency category calculated individually per breed. CAN: Canadian animals; AUS: Australian animals. (DOCX 16 kb) [file 12864_2017_3610_MOESM3_ESM.docx]

**Figure S1.** Percentage of SNP in each allele frequency category calculated individually per breed. **CAN:** Canadian animals; **AUS:** Australian animals.
