# Supplementary figures and images for: Genetic diversity and signatures of selection in various goat breeds revealed by genome-wide SNP markers
Source: BMC Genomics. 2017 Mar 14;18:229. doi: 10.1186/s12864-017-3610-0 (PMC5348779; doi:10.1186/s12864-017-3610-0)

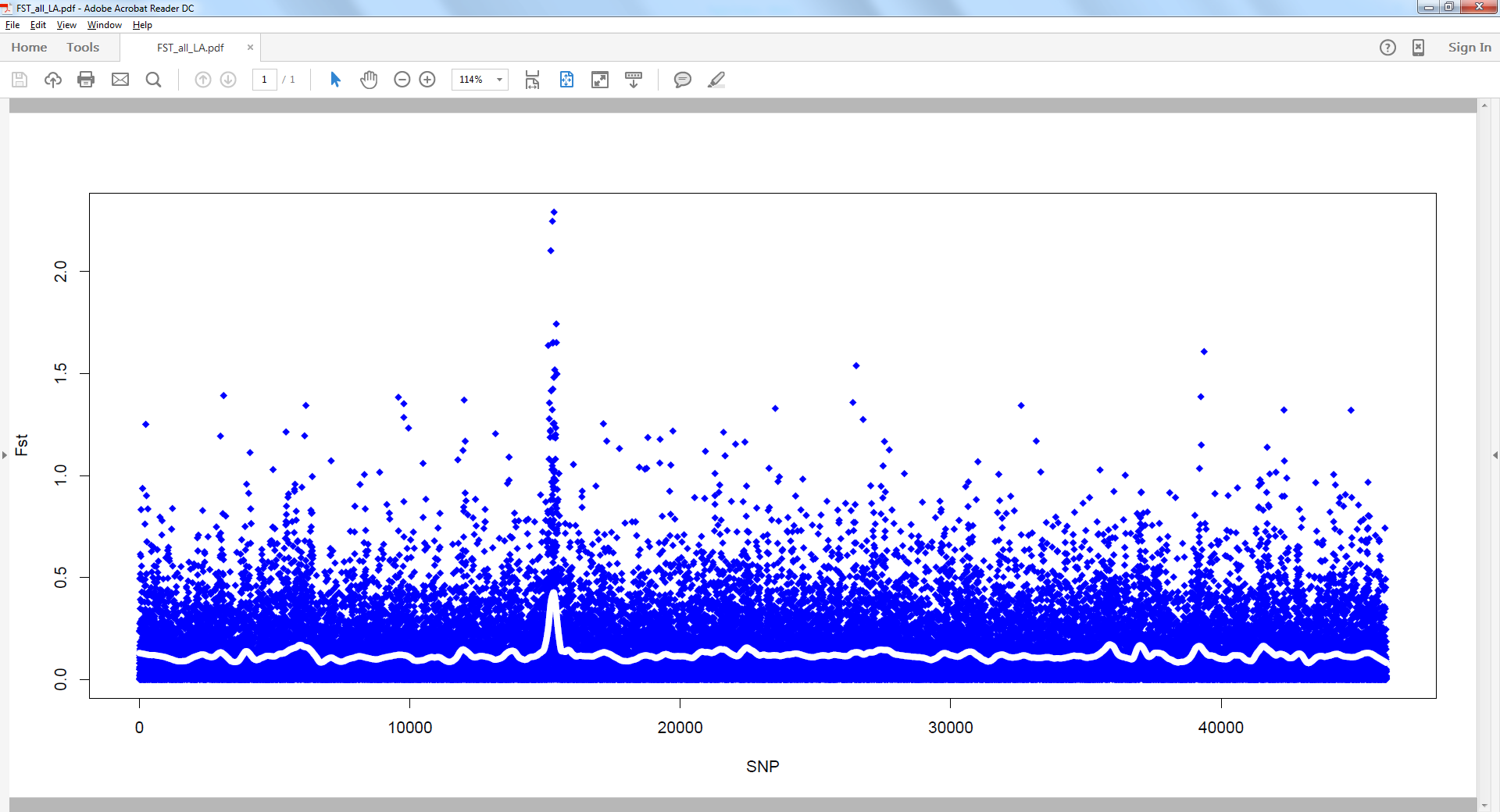


**Figure S1.** FST (blue dots) and smoothed FST (white line) values for the LaMancha breed.

Supplement: Additional file 4: — FST (blue dots) and smoothed FST (white line) values for the LaMancha breed. (DOCX 188 kb) [file 12864_2017_3610_MOESM4_ESM.docx]

**Smoothed FST – Scenario 1 (FST1)**
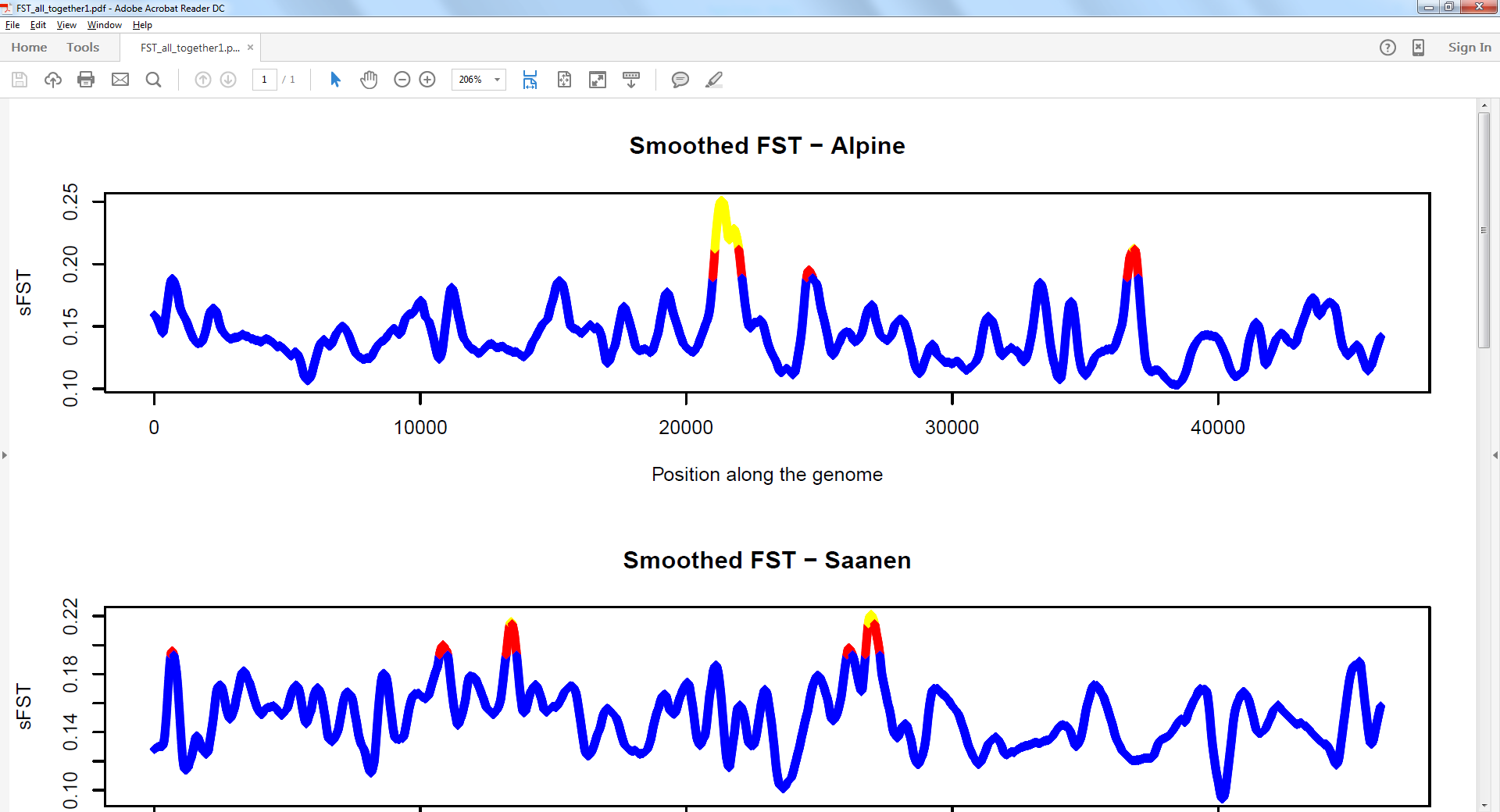

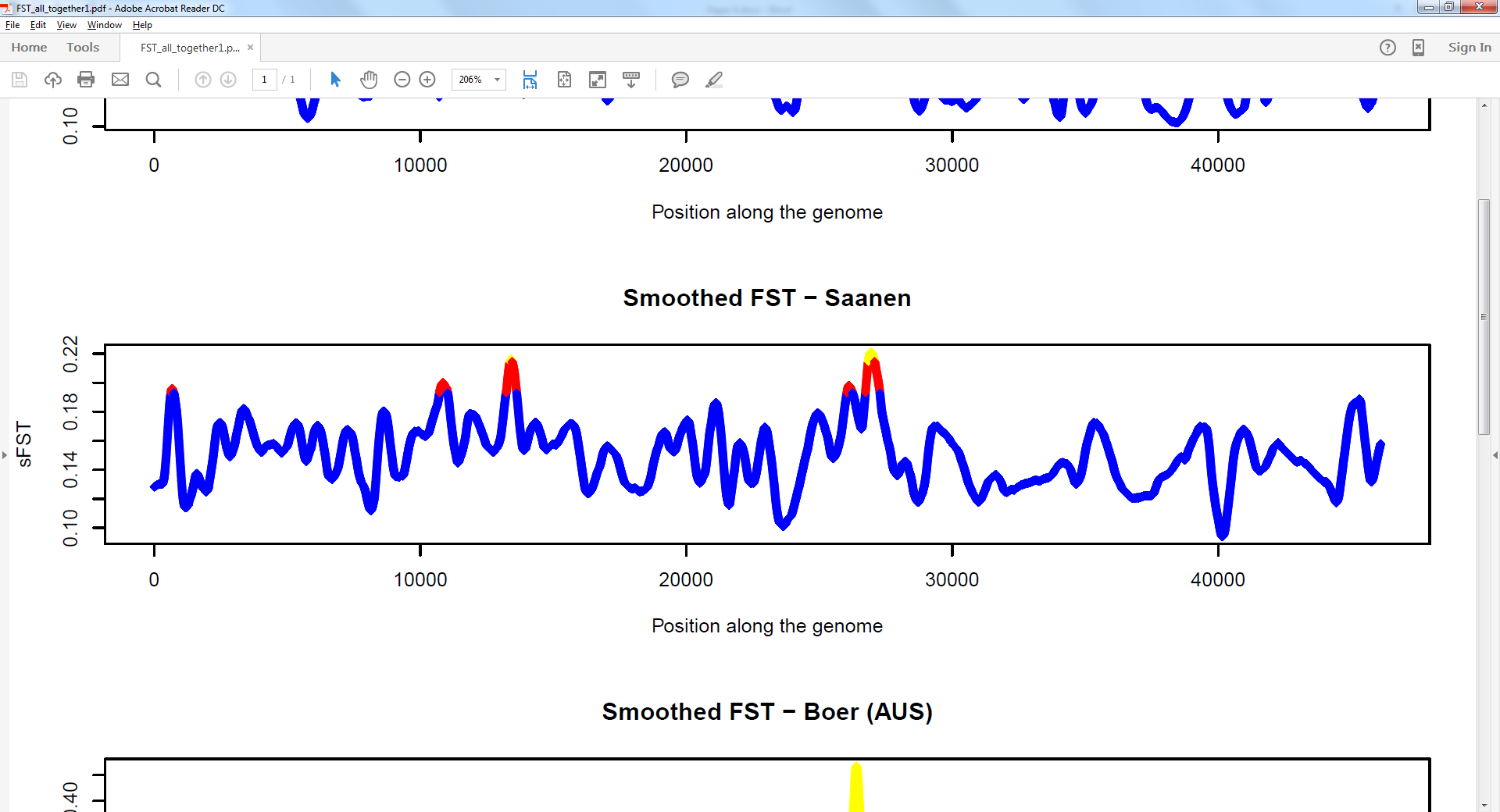


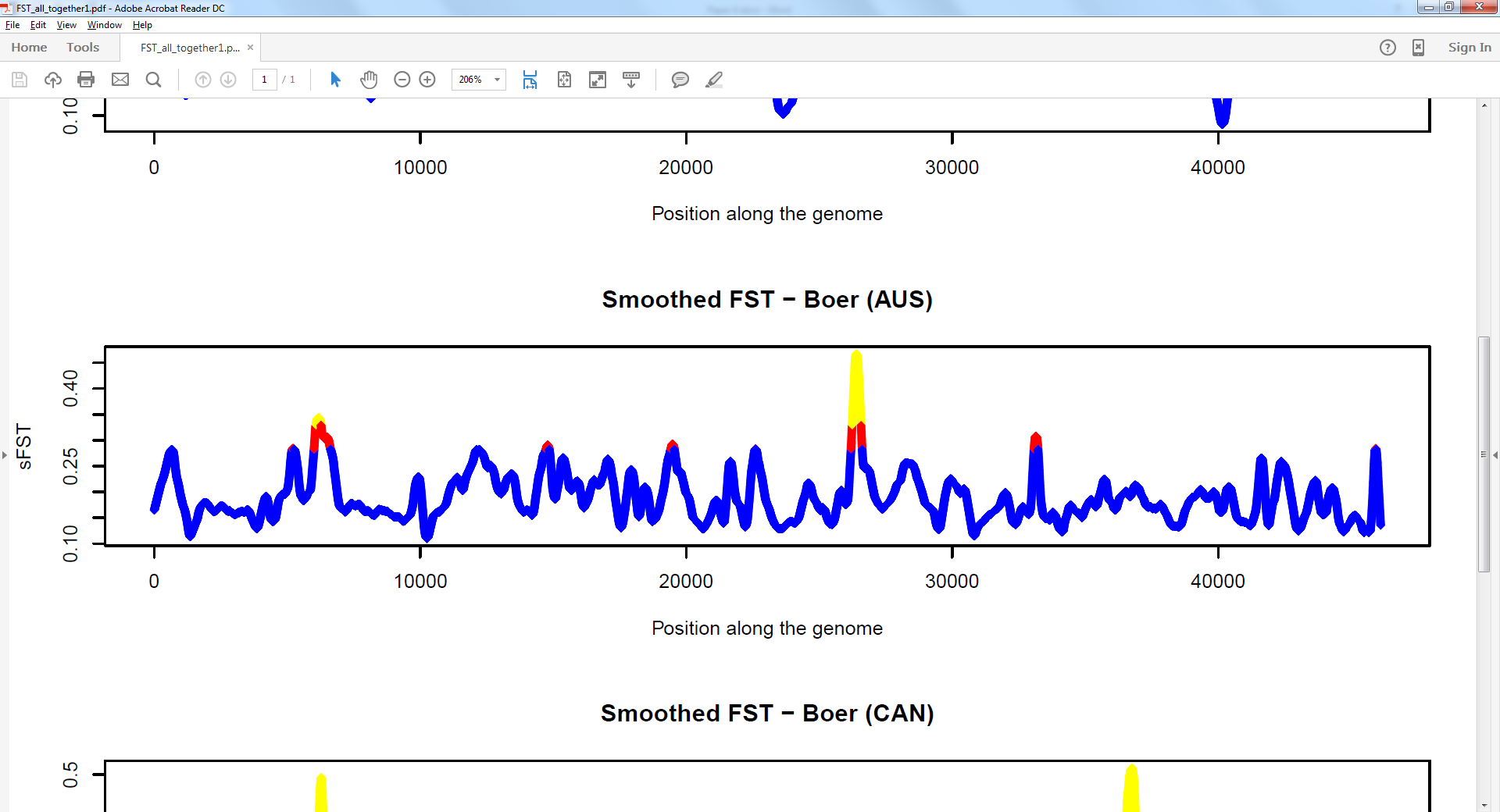


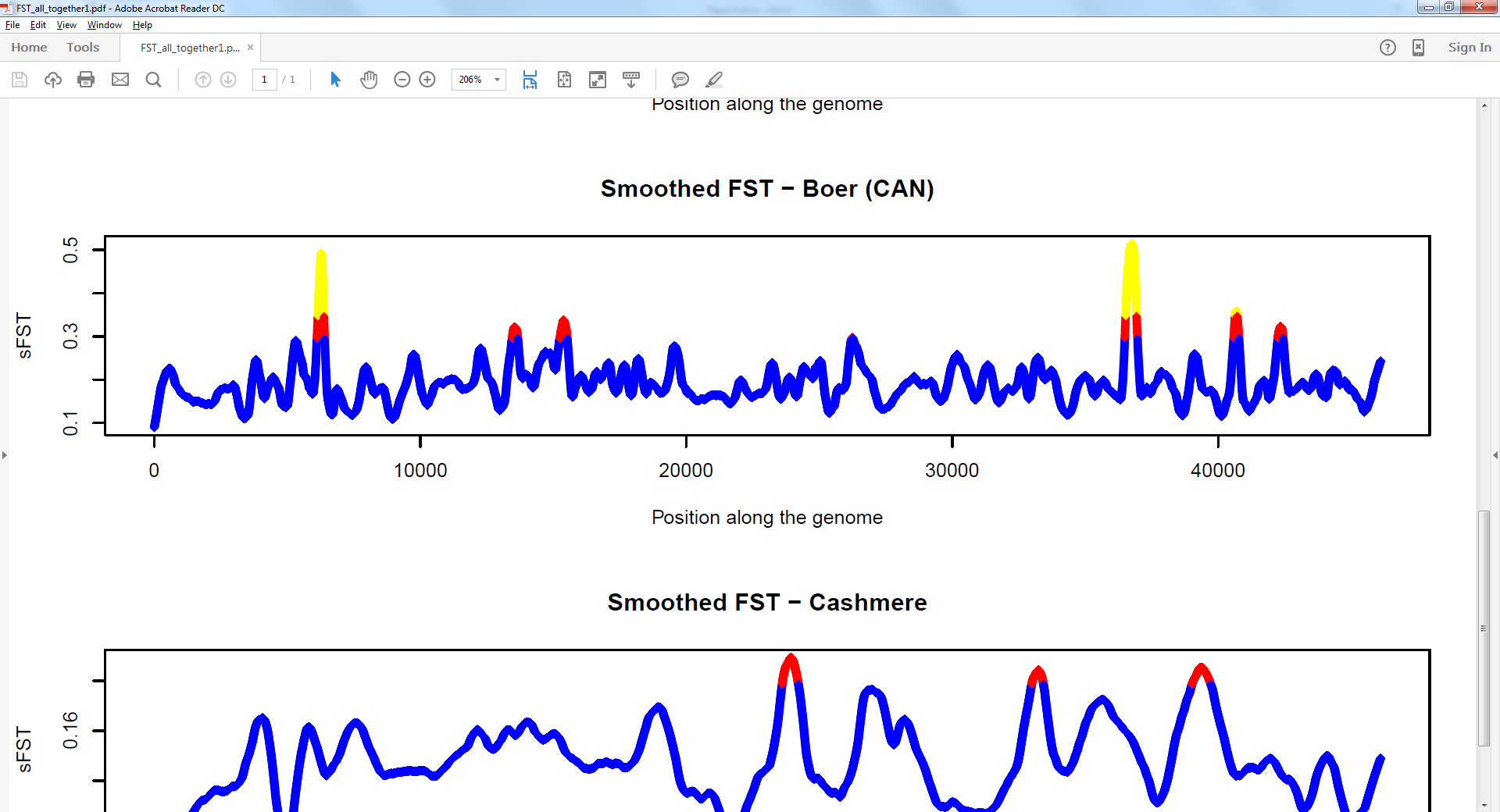


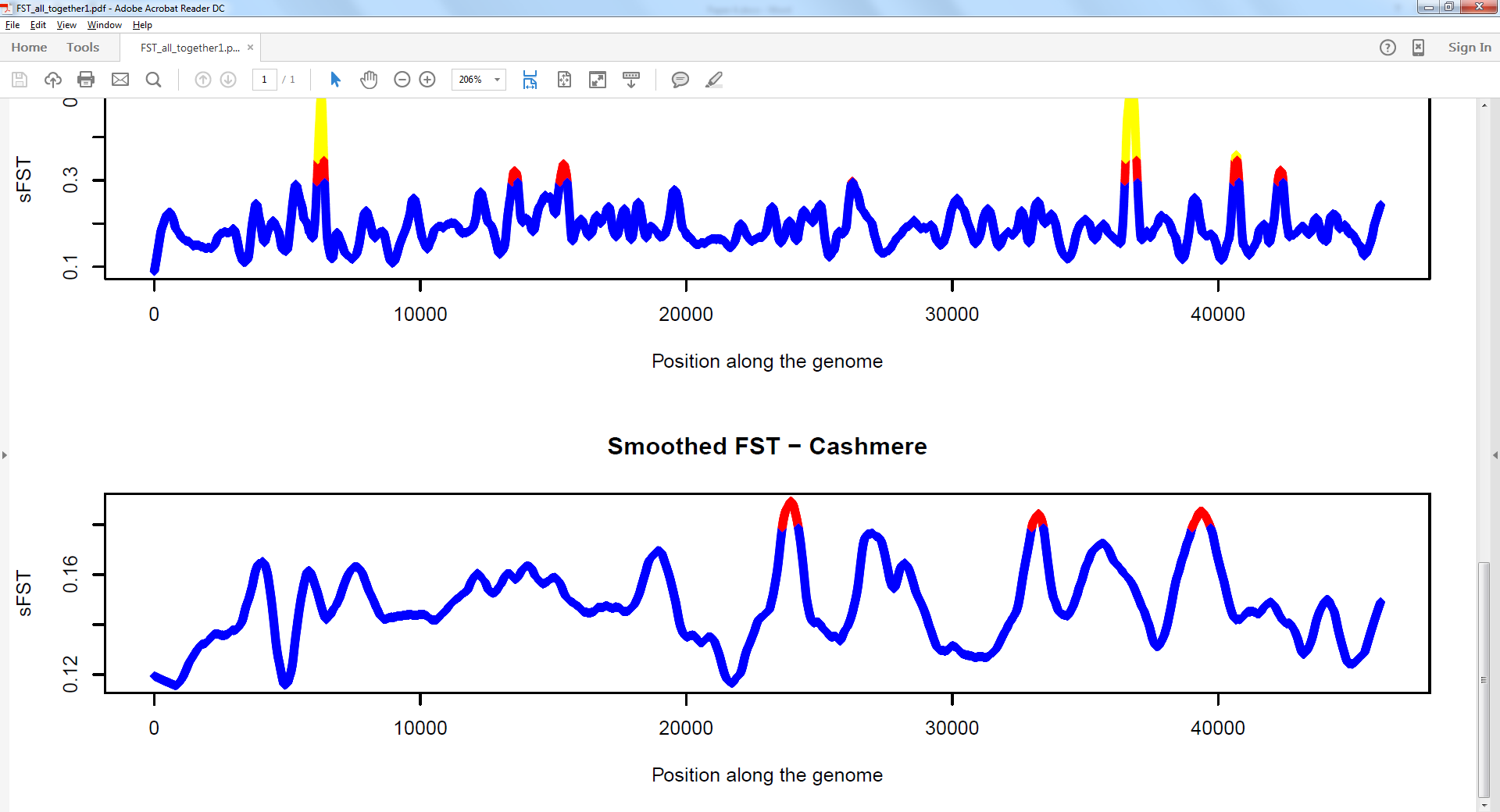


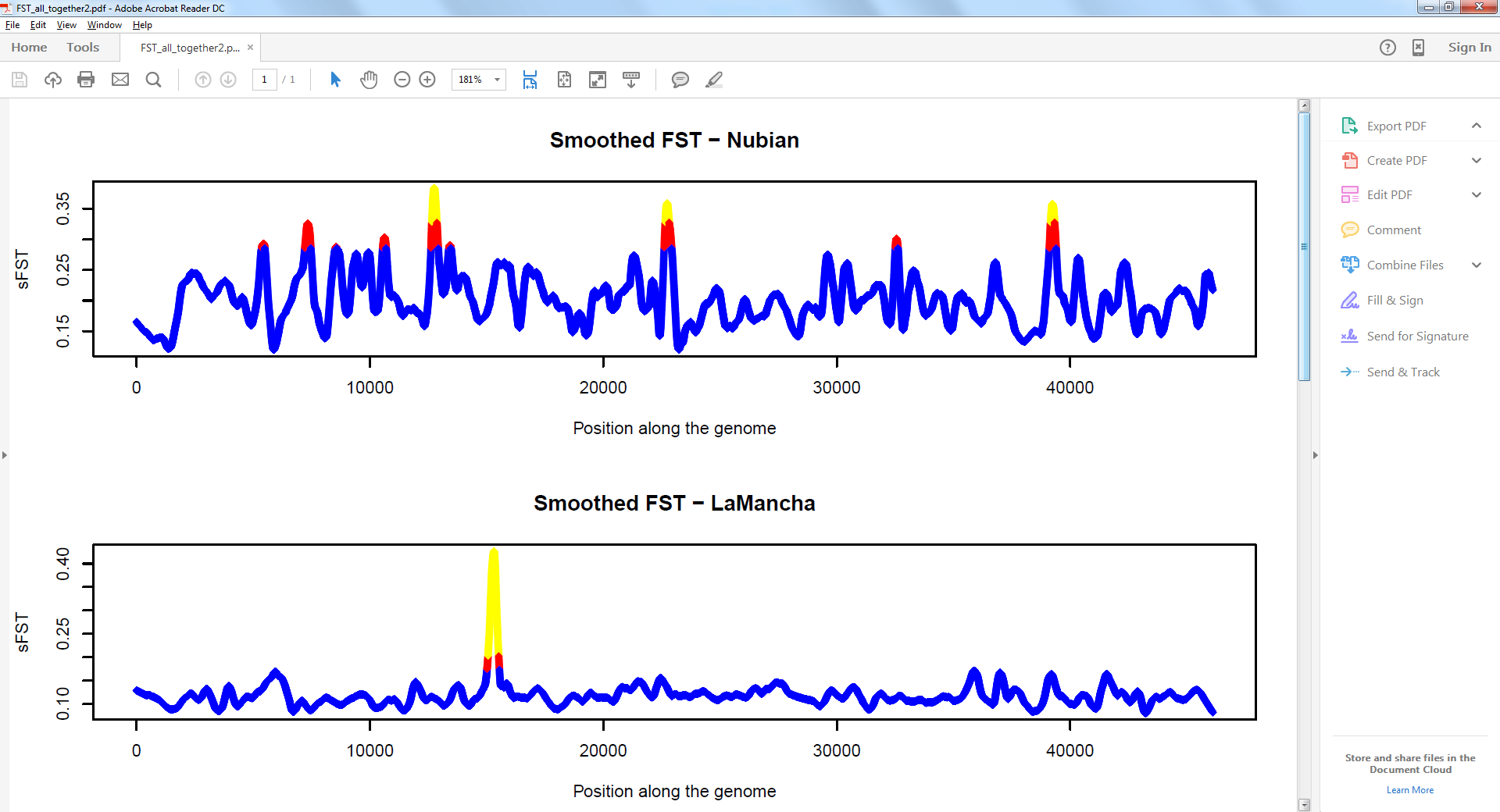


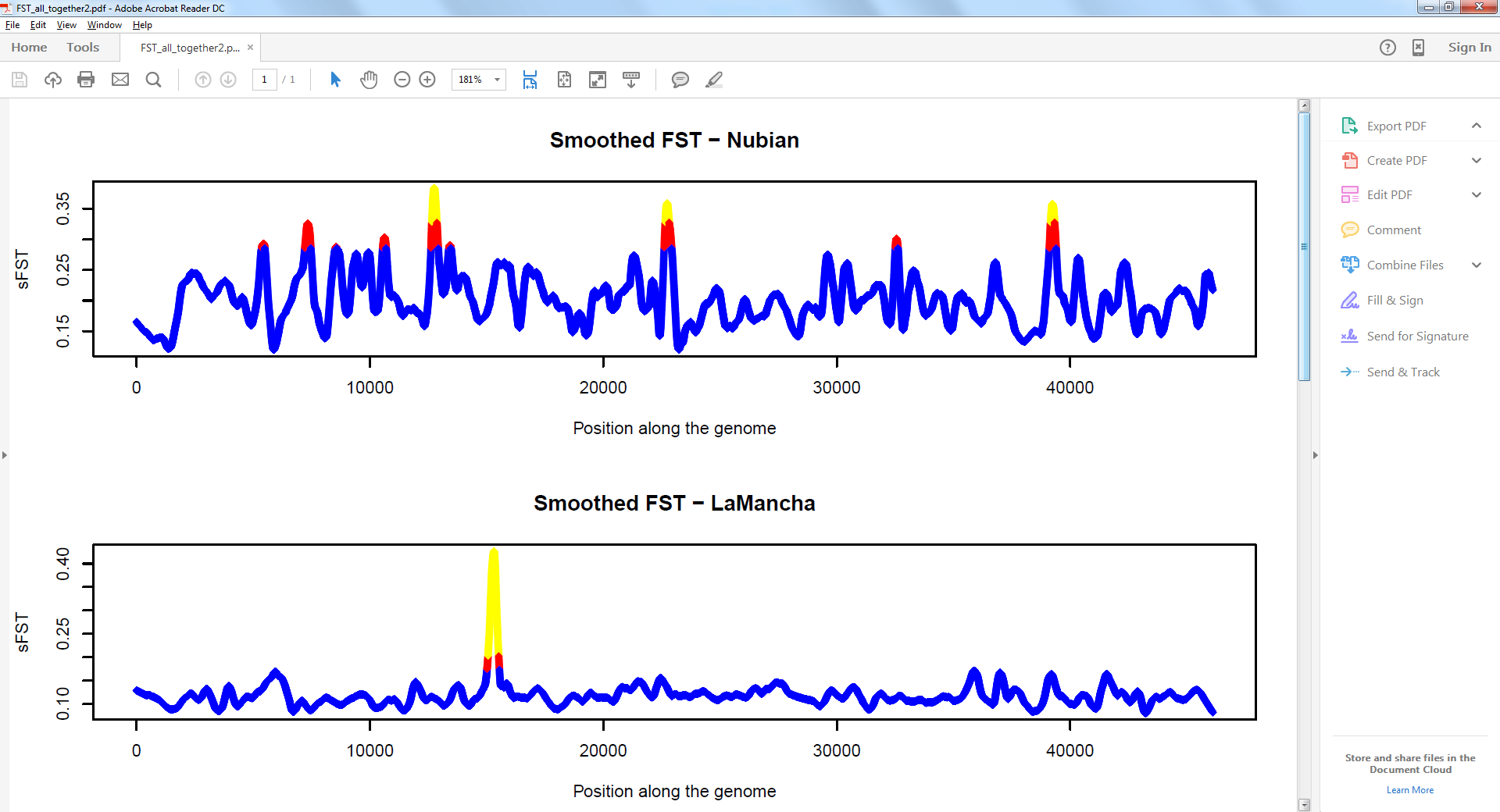


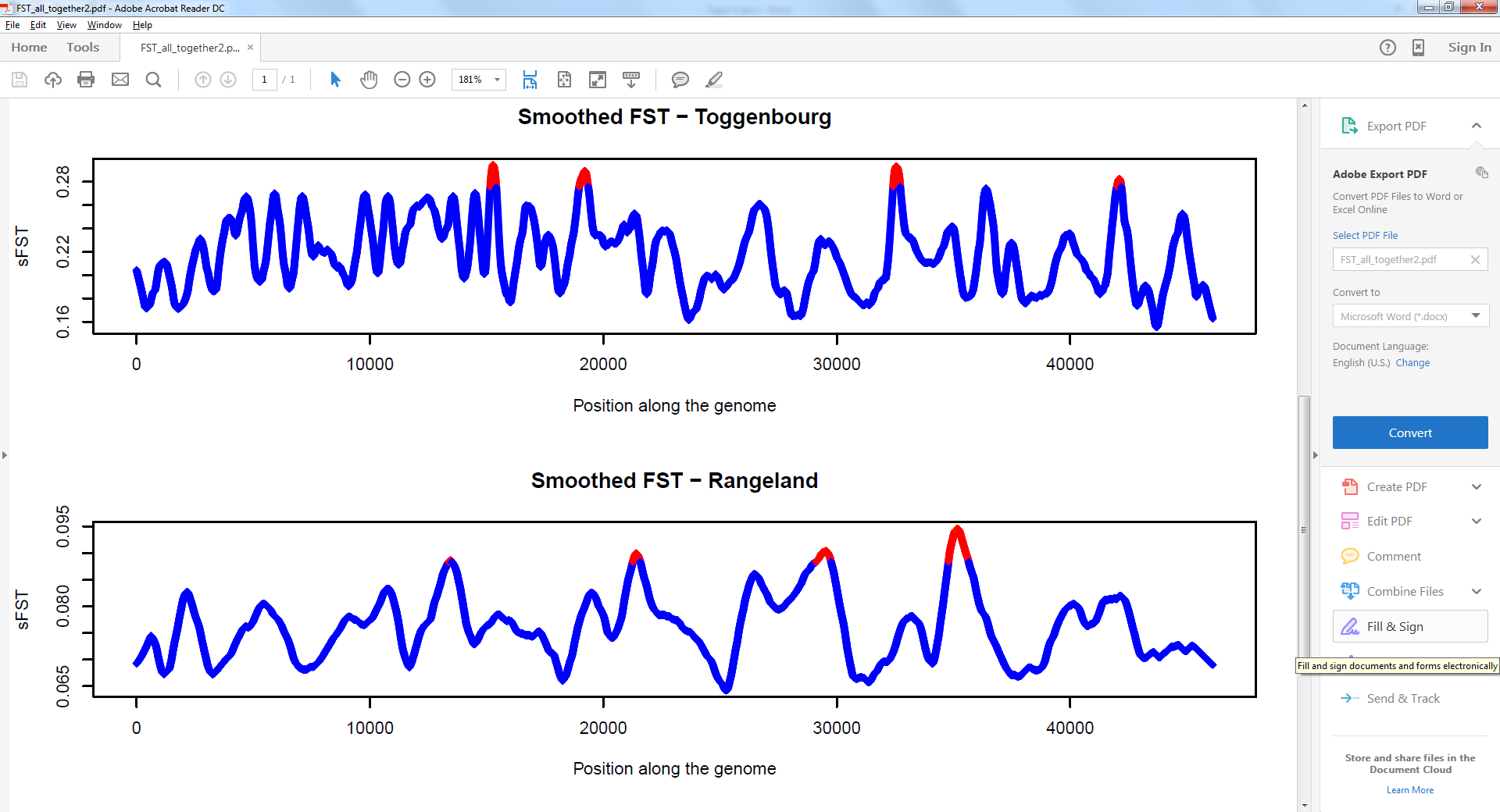


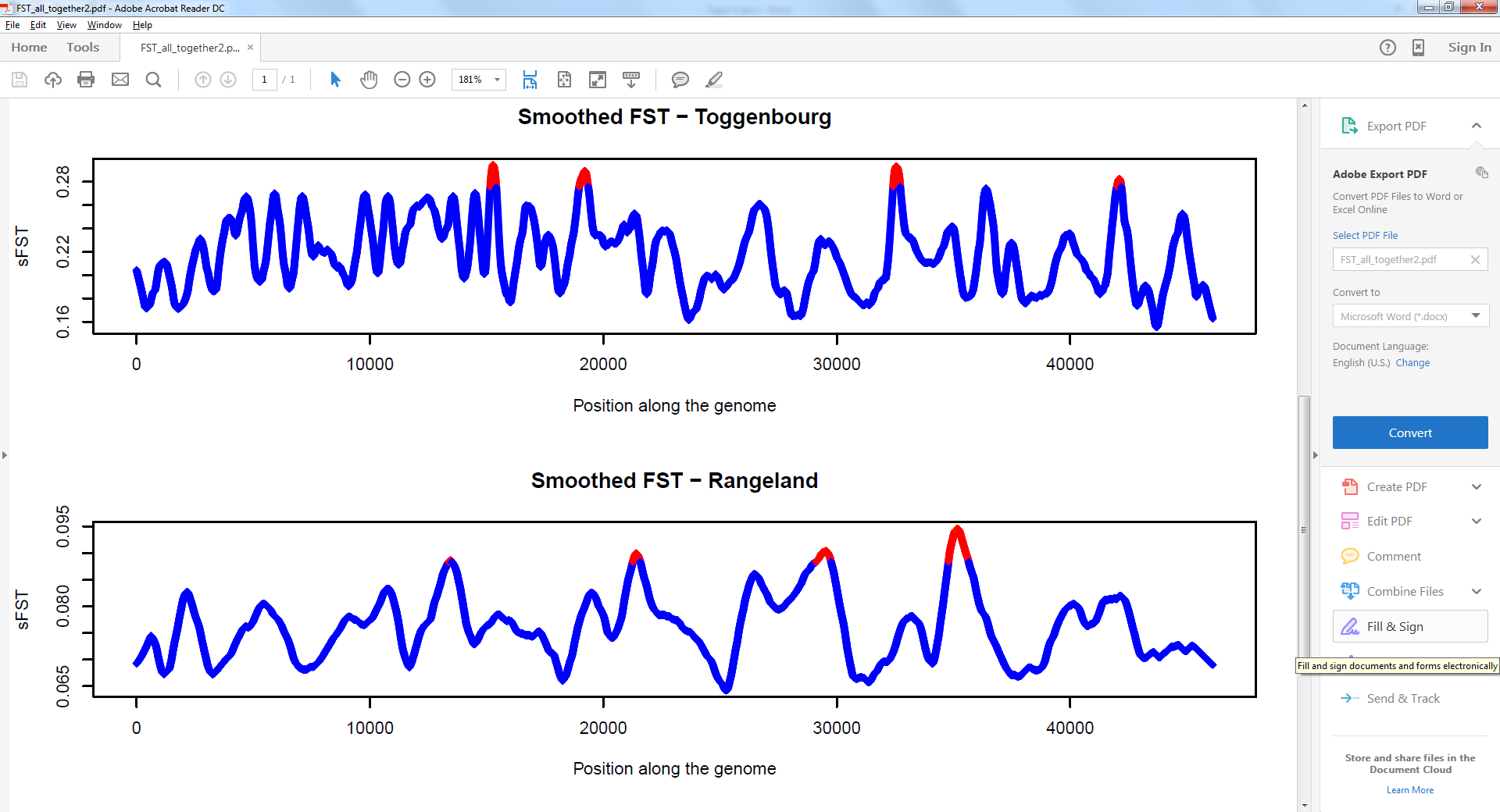


**Smoothed FST - Scenario 2 (FST2):**


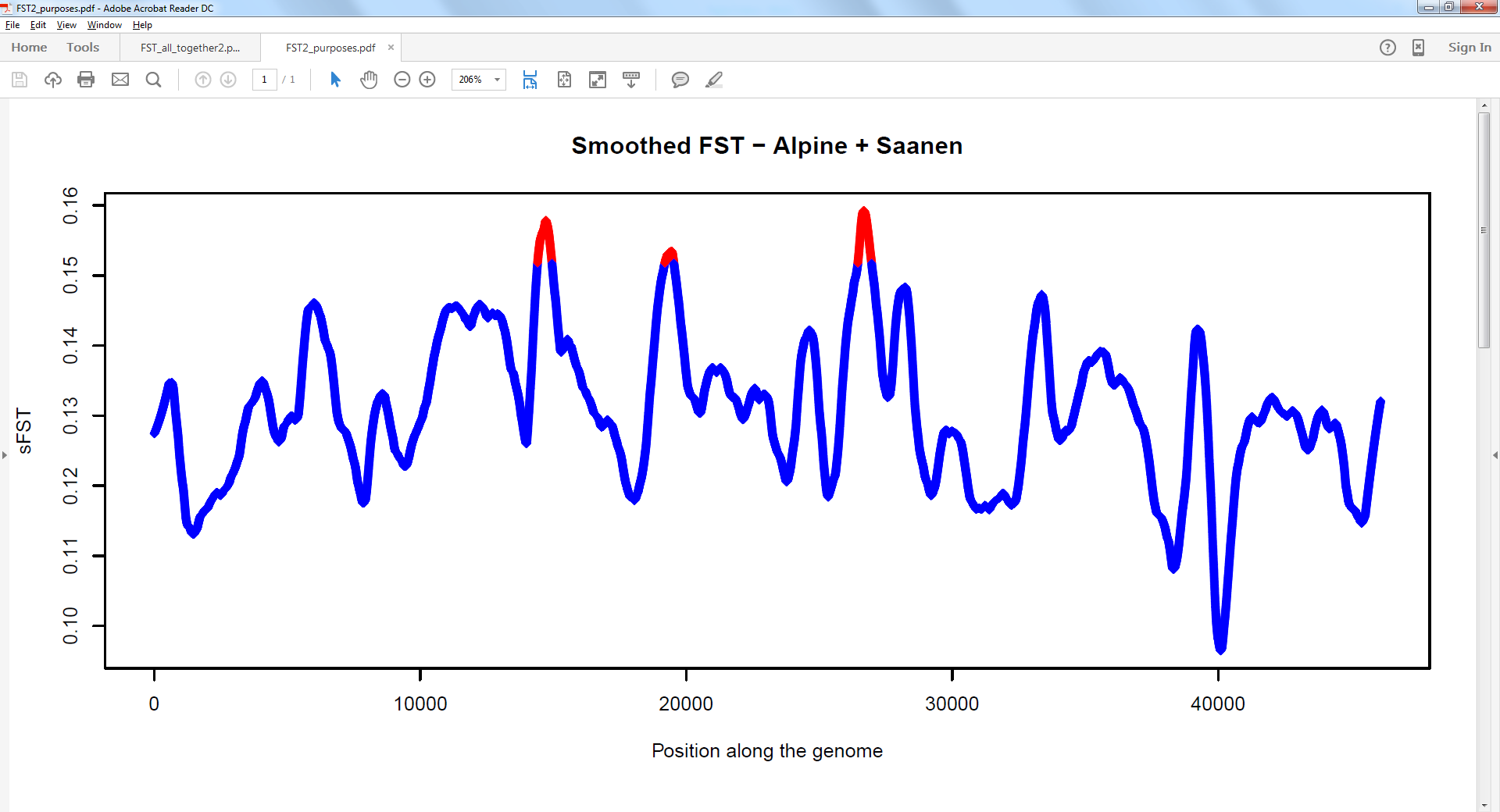


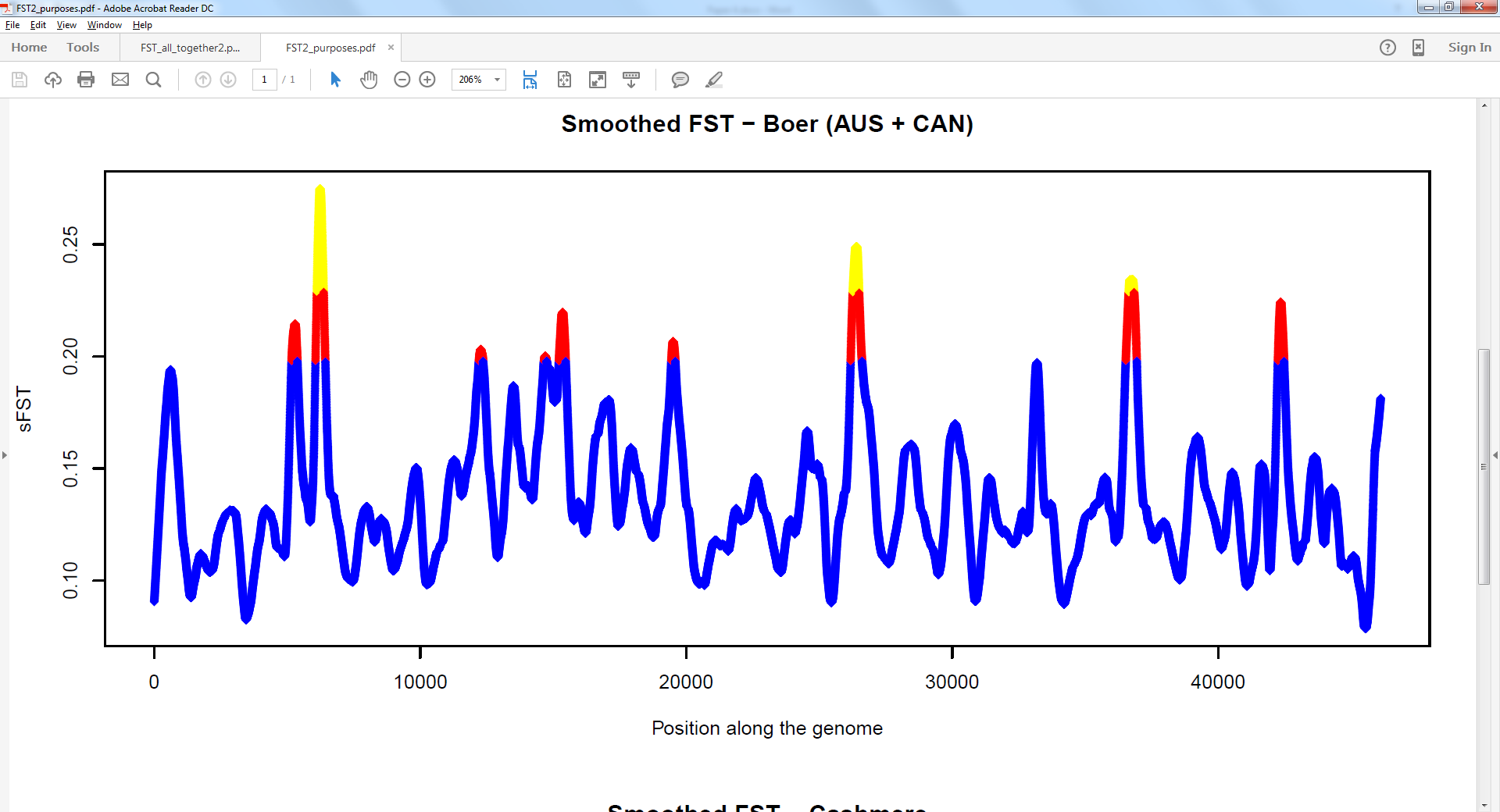


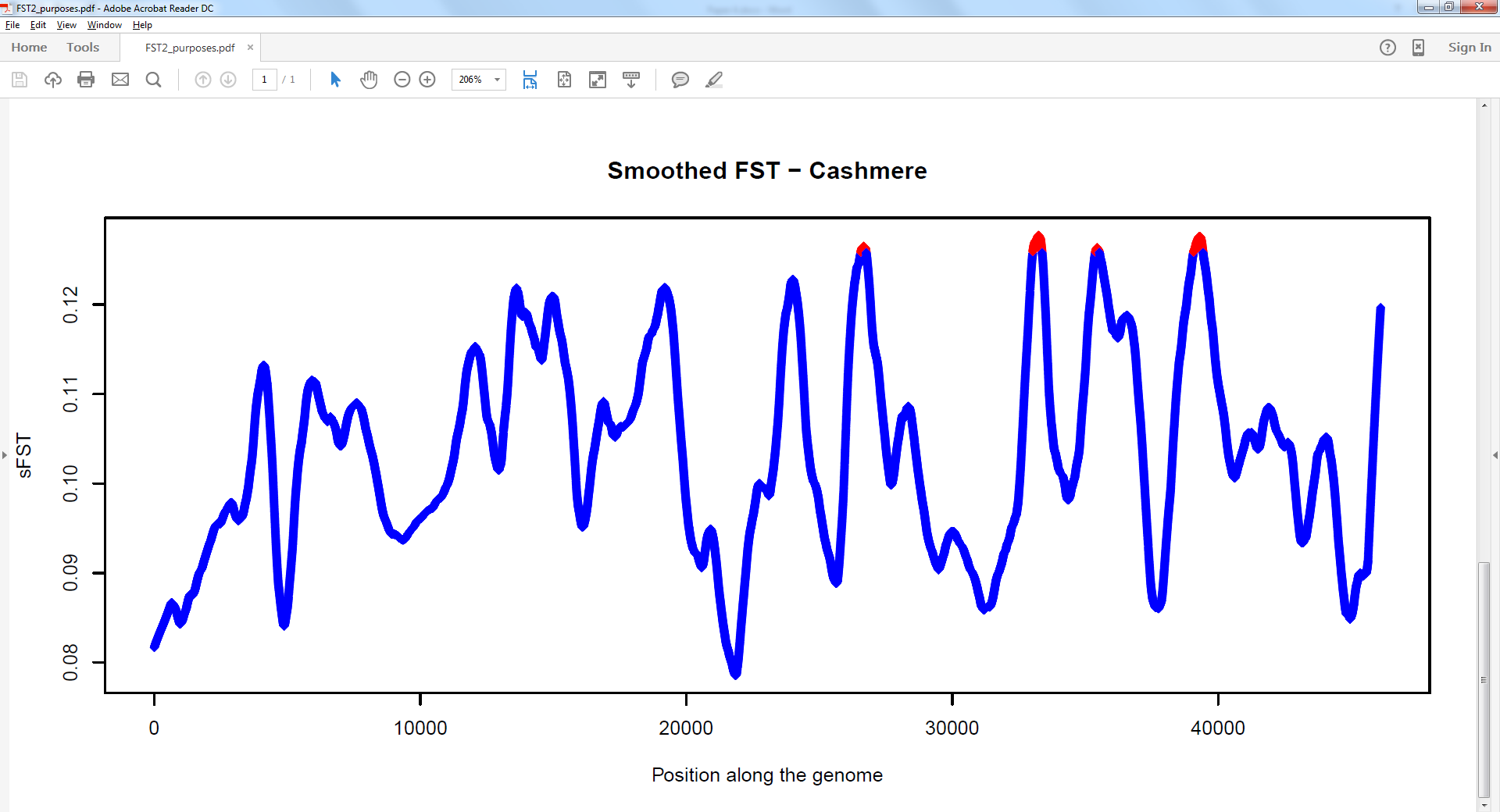


**Smoothed FST- Scenario 3 (FST3):**


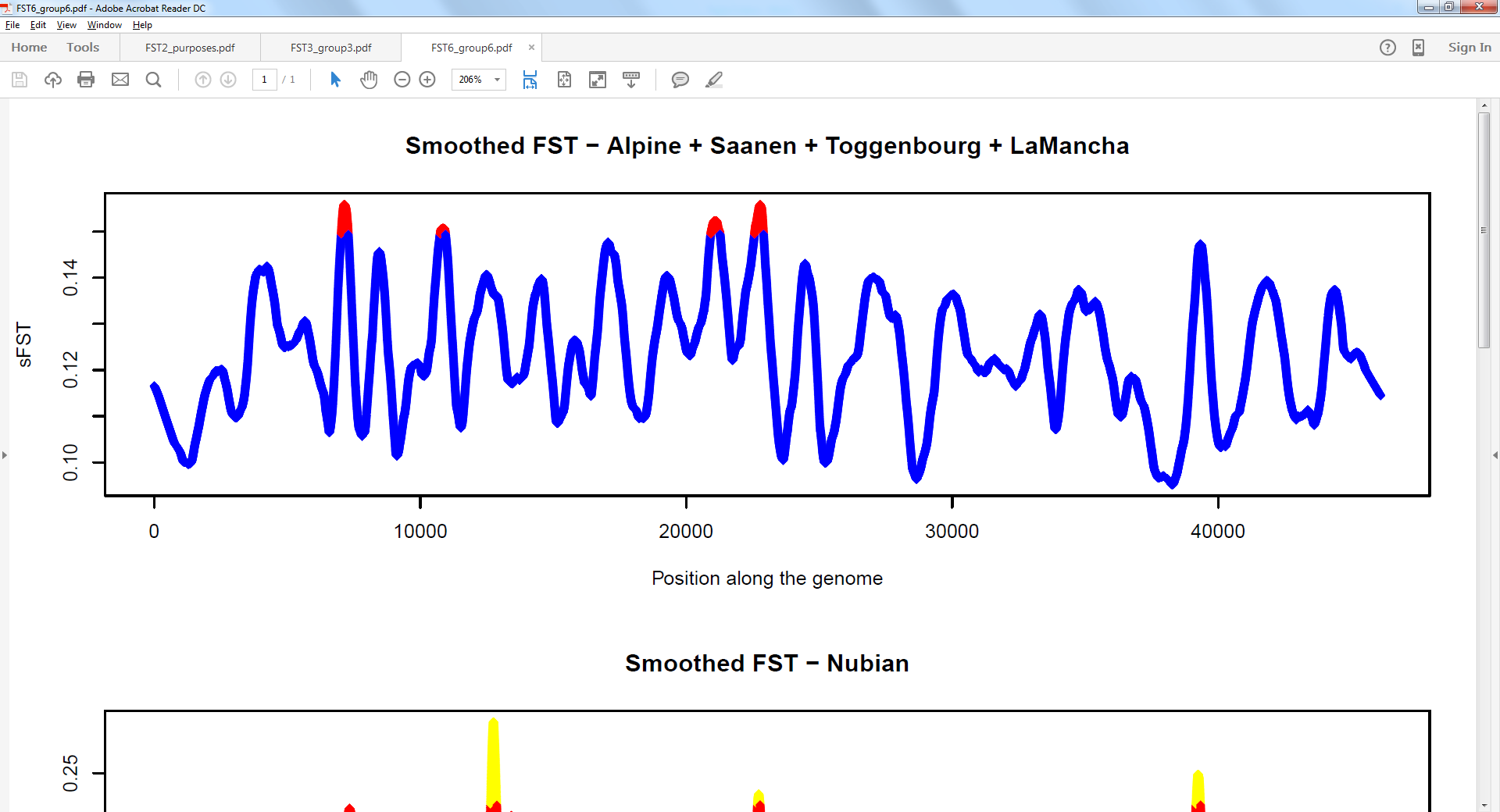


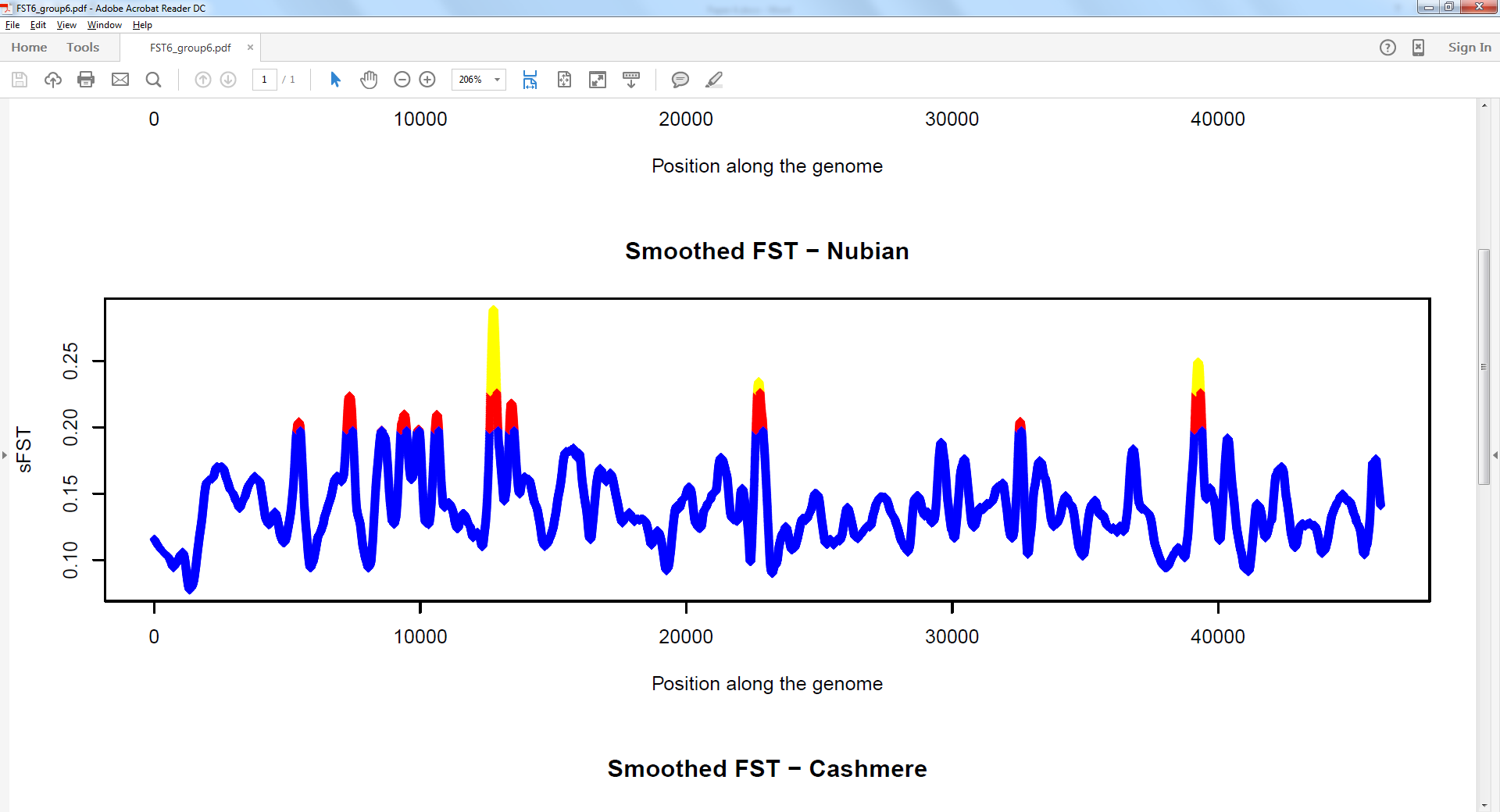


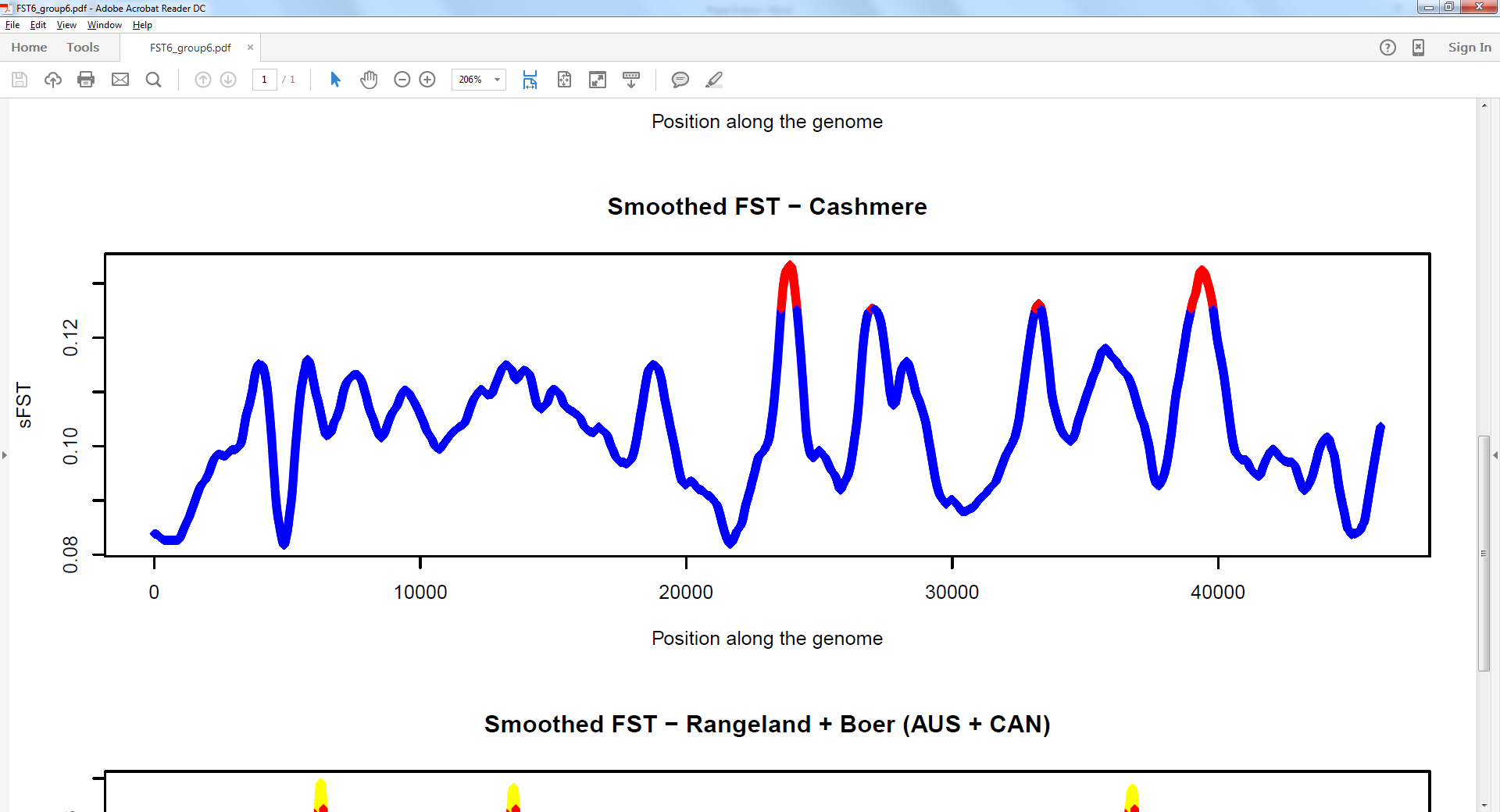


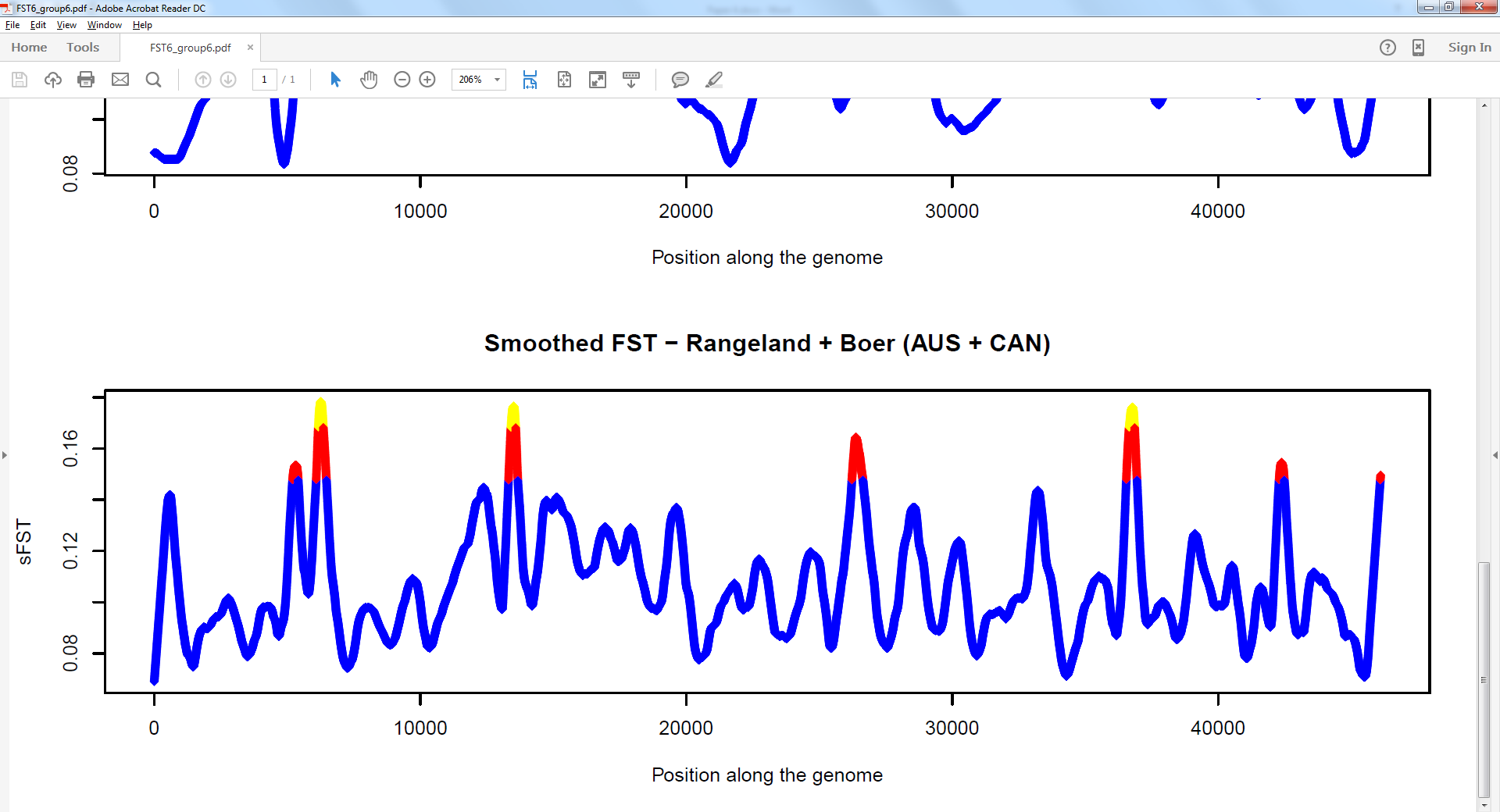

Supplement: Additional file 5: — Smoothed FST for all the scenarios investigated and for all the breeds included in this study. (DOCX 2305 kb) [file 12864_2017_3610_MOESM5_ESM.docx]
