# Supplementary material for: Genetic diversity and signatures of selection in various goat breeds revealed by genome-wide SNP markers
Source: BMC Genomics. 2017 Mar 14;18:229. doi: 10.1186/s12864-017-3610-0 (PMC5348779; doi:10.1186/s12864-017-3610-0)
Supplement: Additional file 7: — Whole genome scans for selection using the haplotype based hapFLK metric and –log (P-values) were plotted in genomic order only for the chromosome 7 (highest peaks). SNP number is given on the x axis, and the genome-wide threshold corresponding to P < 0.001, P < 0.005 and P < 0.01 is shown as horizontal blue, green and red lines, respectively. (DOCX 124 kb) [file 12864_2017_3610_MOESM7_ESM.docx]

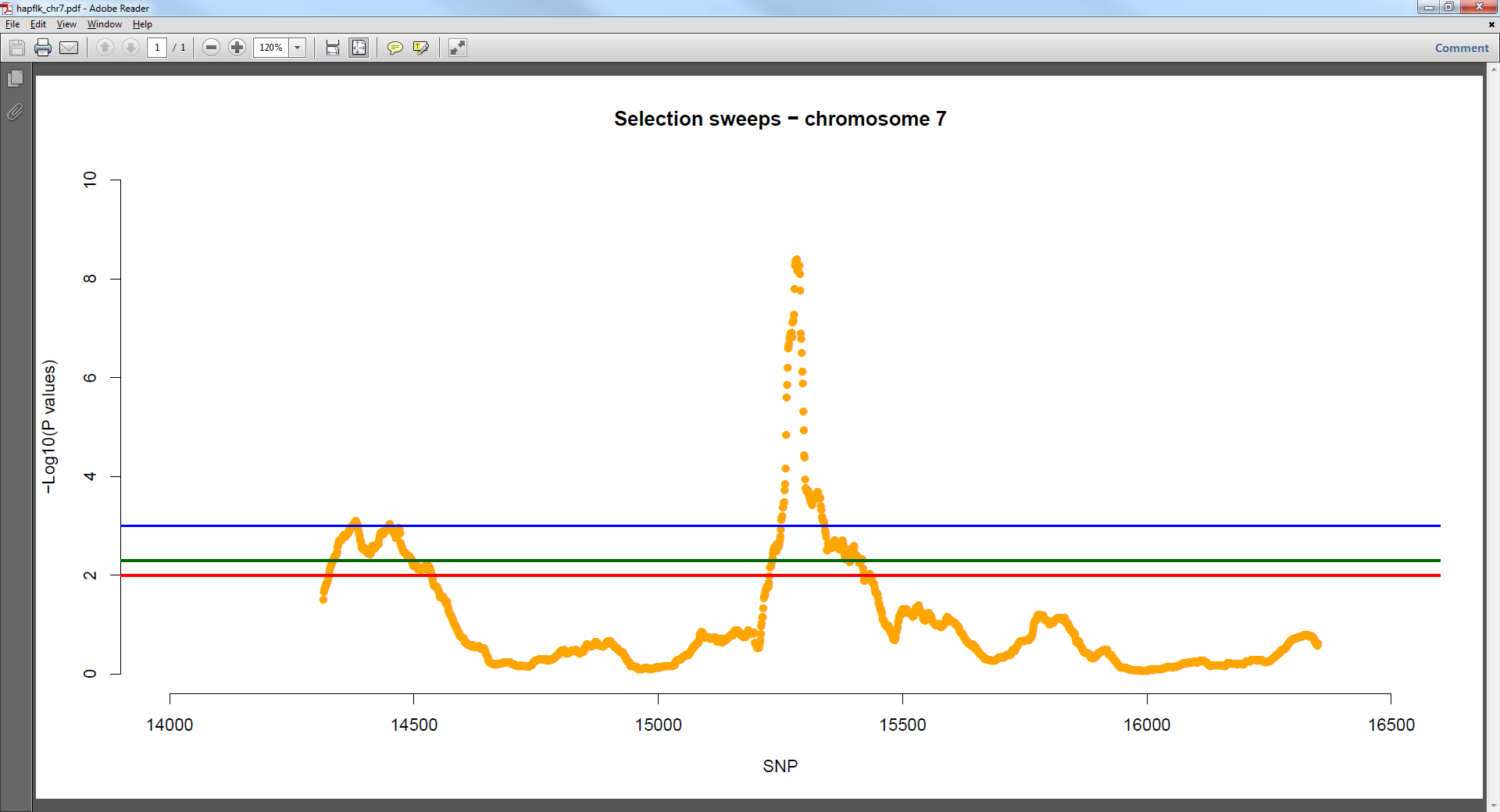


**Figure S1.** Whole genome scans for selection using the haplotype based hapFLK metric and –log (P-values) were plotted in genomic order only for the chromosome 7 (highest peaks). SNP number is given on the x axis, and the genome-wide threshold corresponding to P < 0.001, P < 0.005 and P < 0.01 is shown as horizontal blue, green and red lines, respectively.
