# Supplementary material for: Genetic diversity and signatures of selection in various goat breeds revealed by genome-wide SNP markers
Source: BMC Genomics. 2017 Mar 14;18:229. doi: 10.1186/s12864-017-3610-0 (PMC5348779; doi:10.1186/s12864-017-3610-0)
Supplement: Additional file 8: — Population tree using significant SNPs for each signature of selection region. (DOCX 801 kb) [file 12864_2017_3610_MOESM8_ESM.docx]

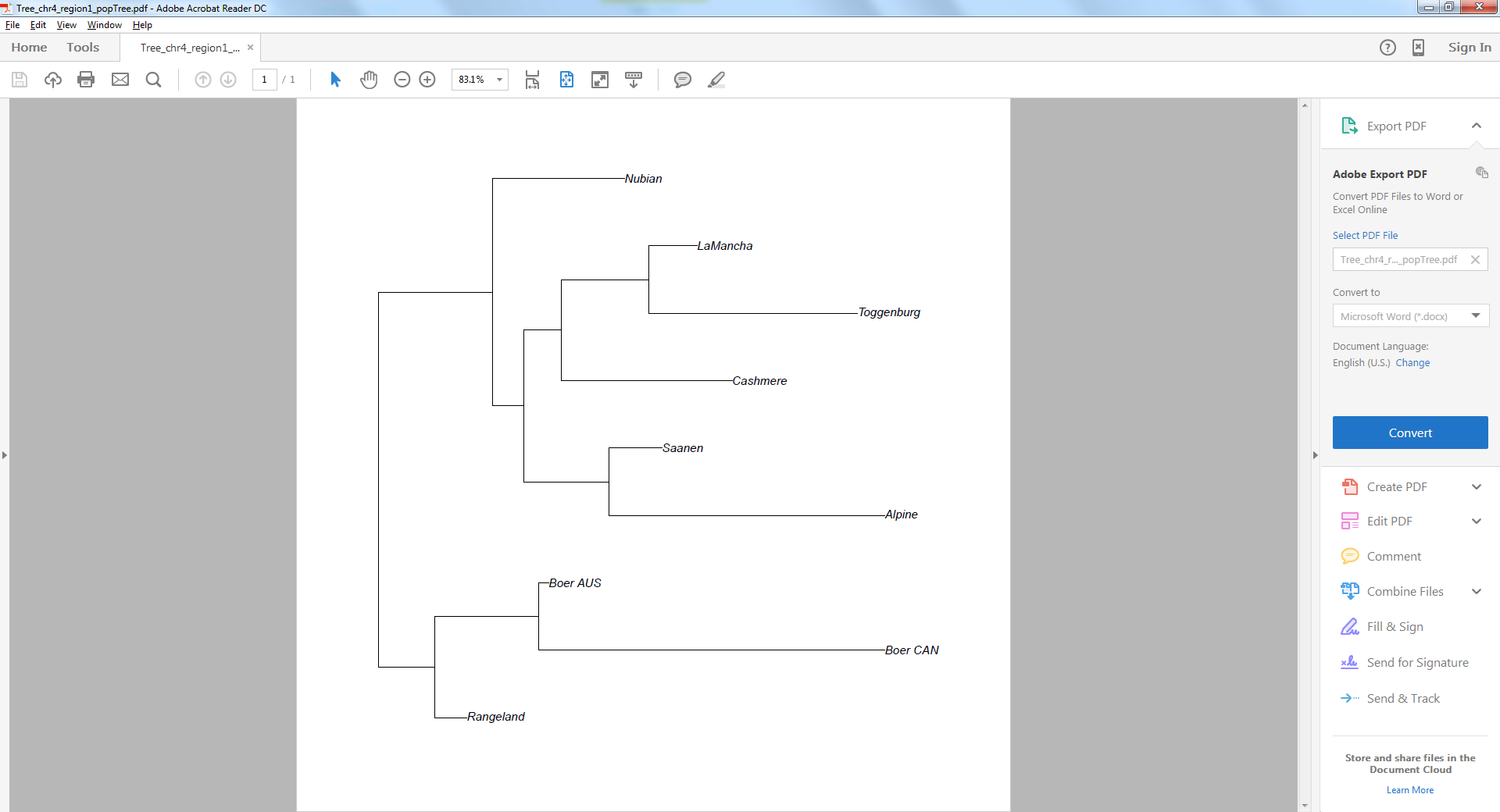


**Figure S1.** Population tree using significant SNPs for region 1, chromosome 4.


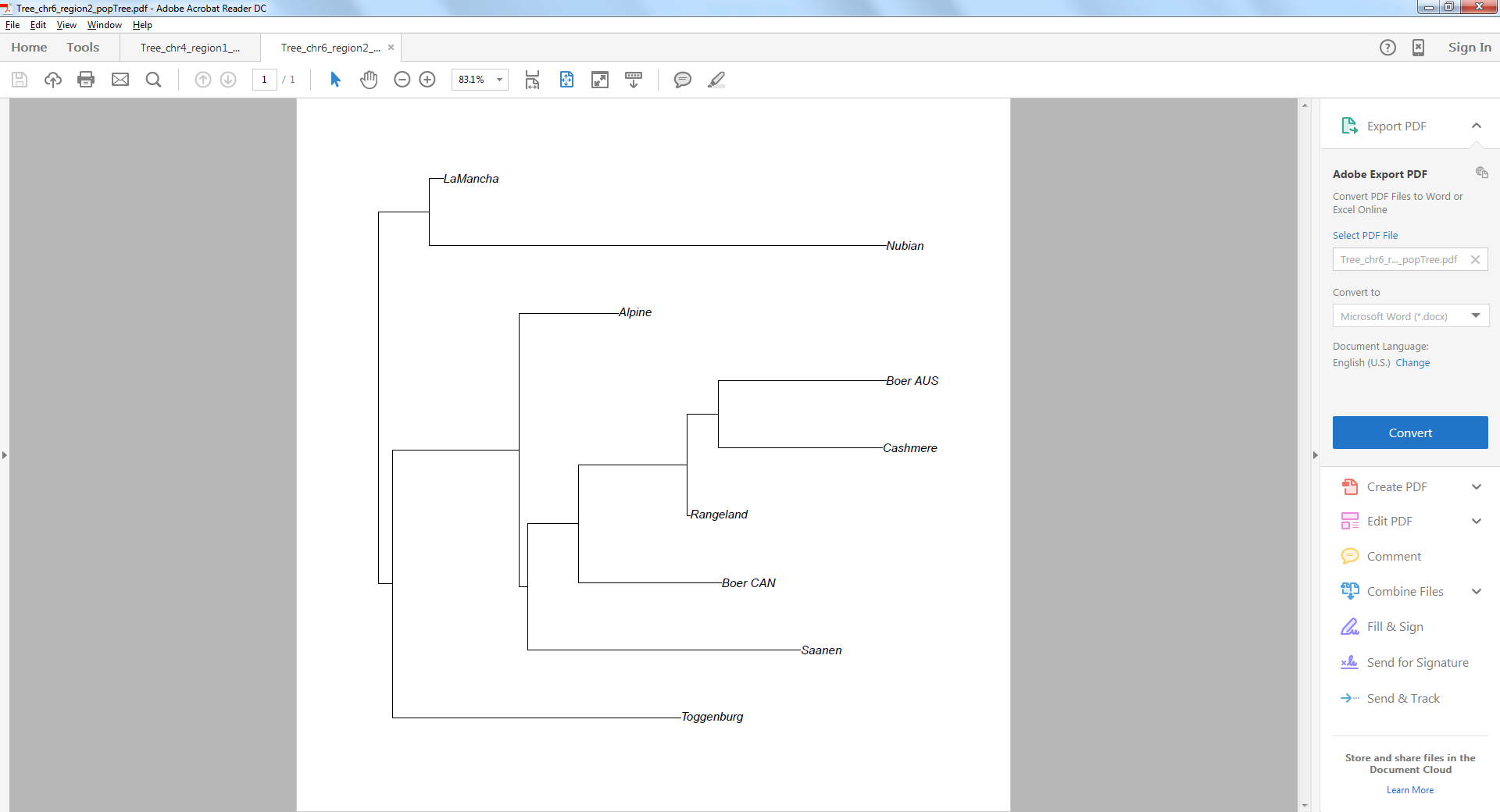


**Figure S2.** Population tree using significant SNPs for region 2, chromosome 6.


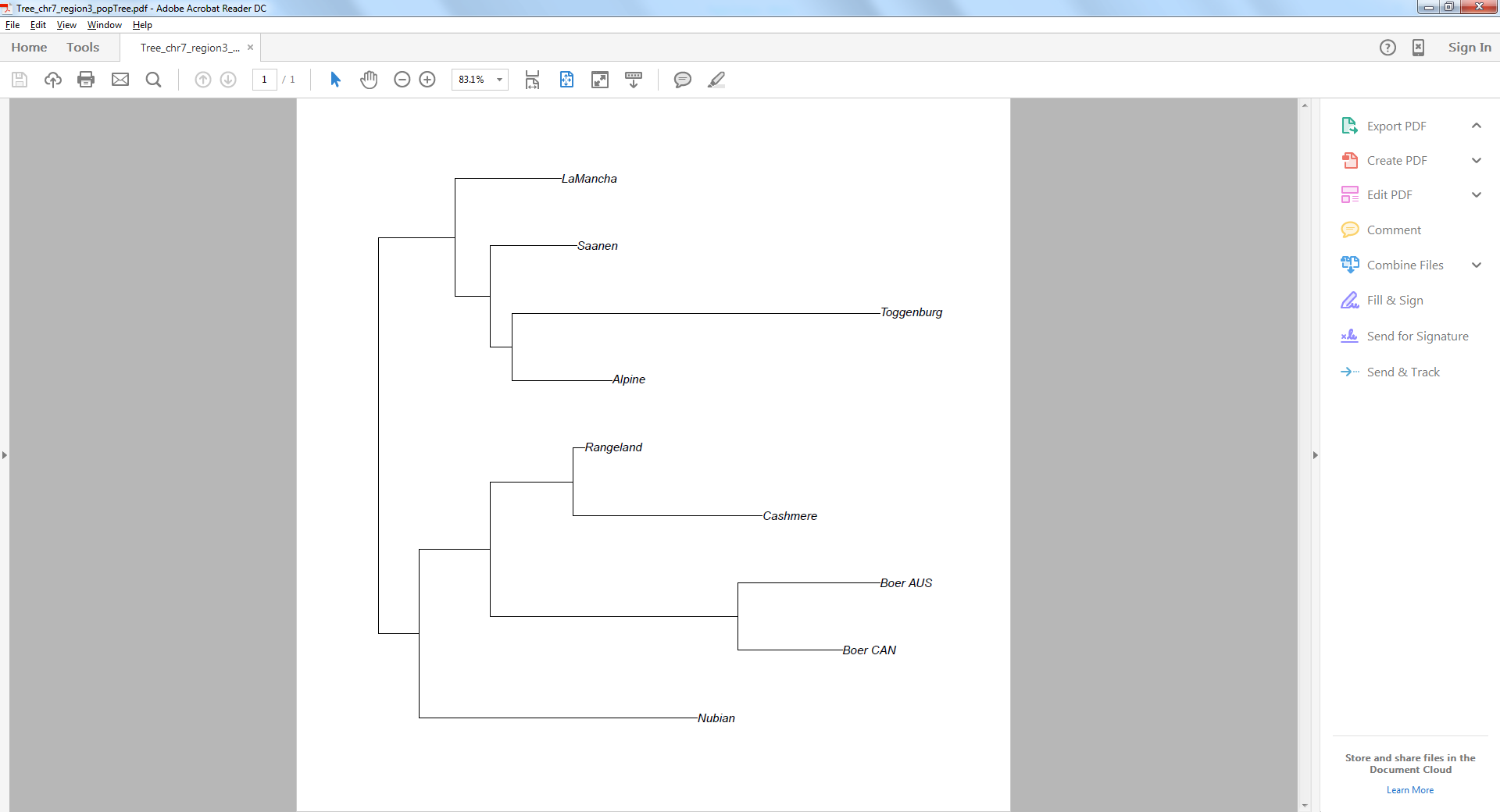


**Figure S3.** Population tree using significant SNPs for region 3, chromosome 7.


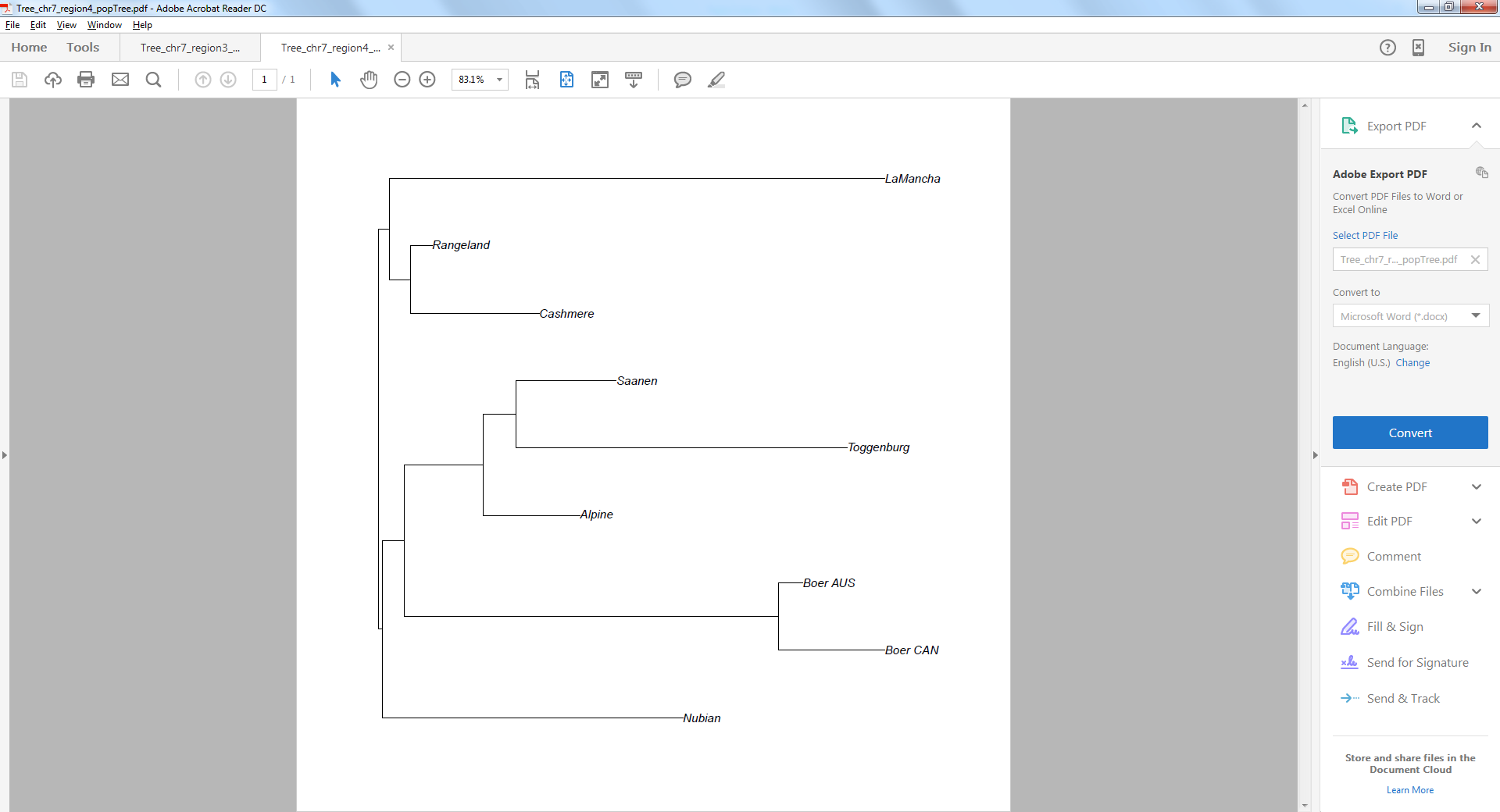


**Figure S4.** Population tree using significant SNPs for region 4, chromosome 7.


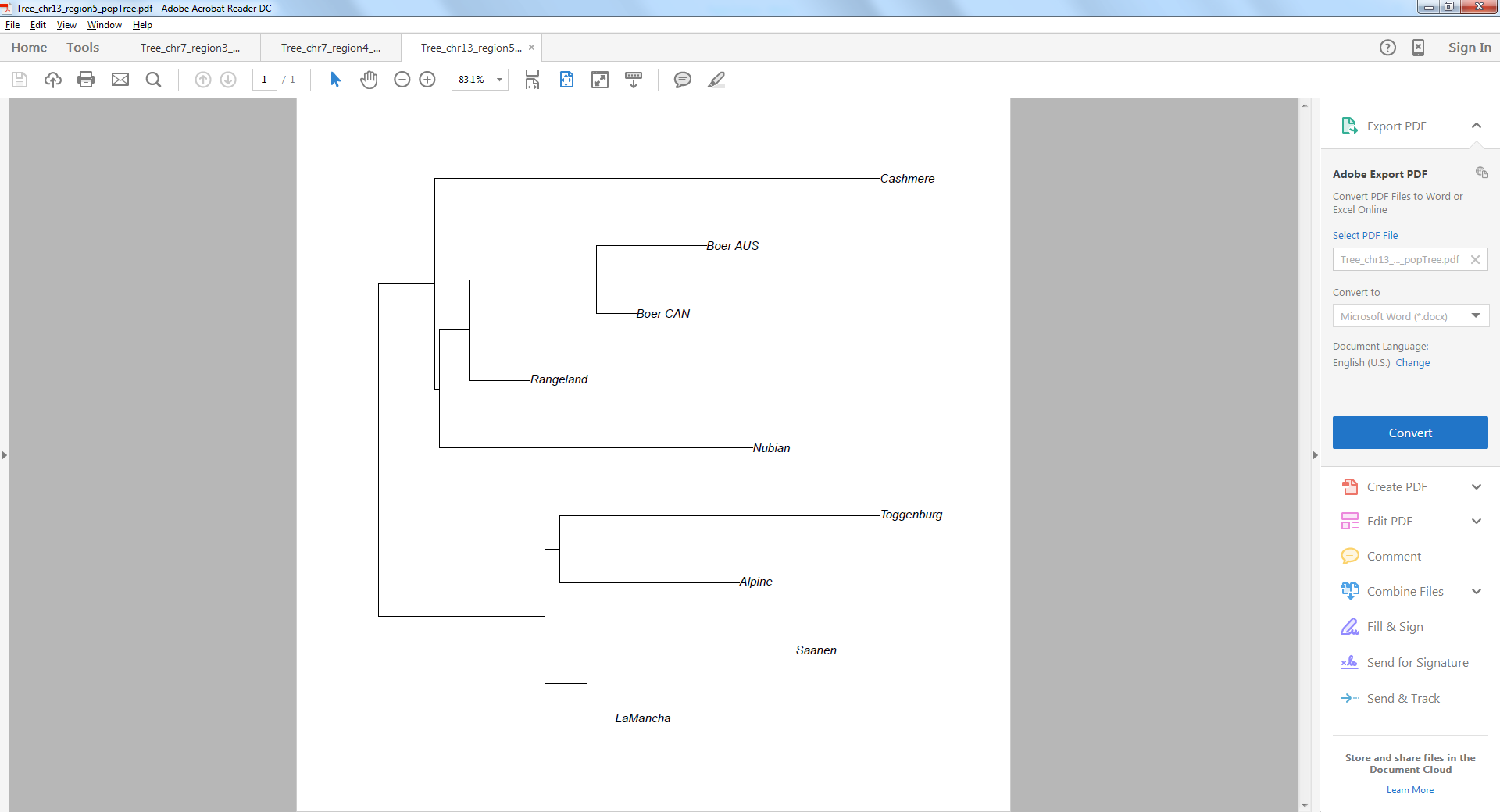


**Figure S5.** Population tree using significant SNPs for region 5, chromosome 13.


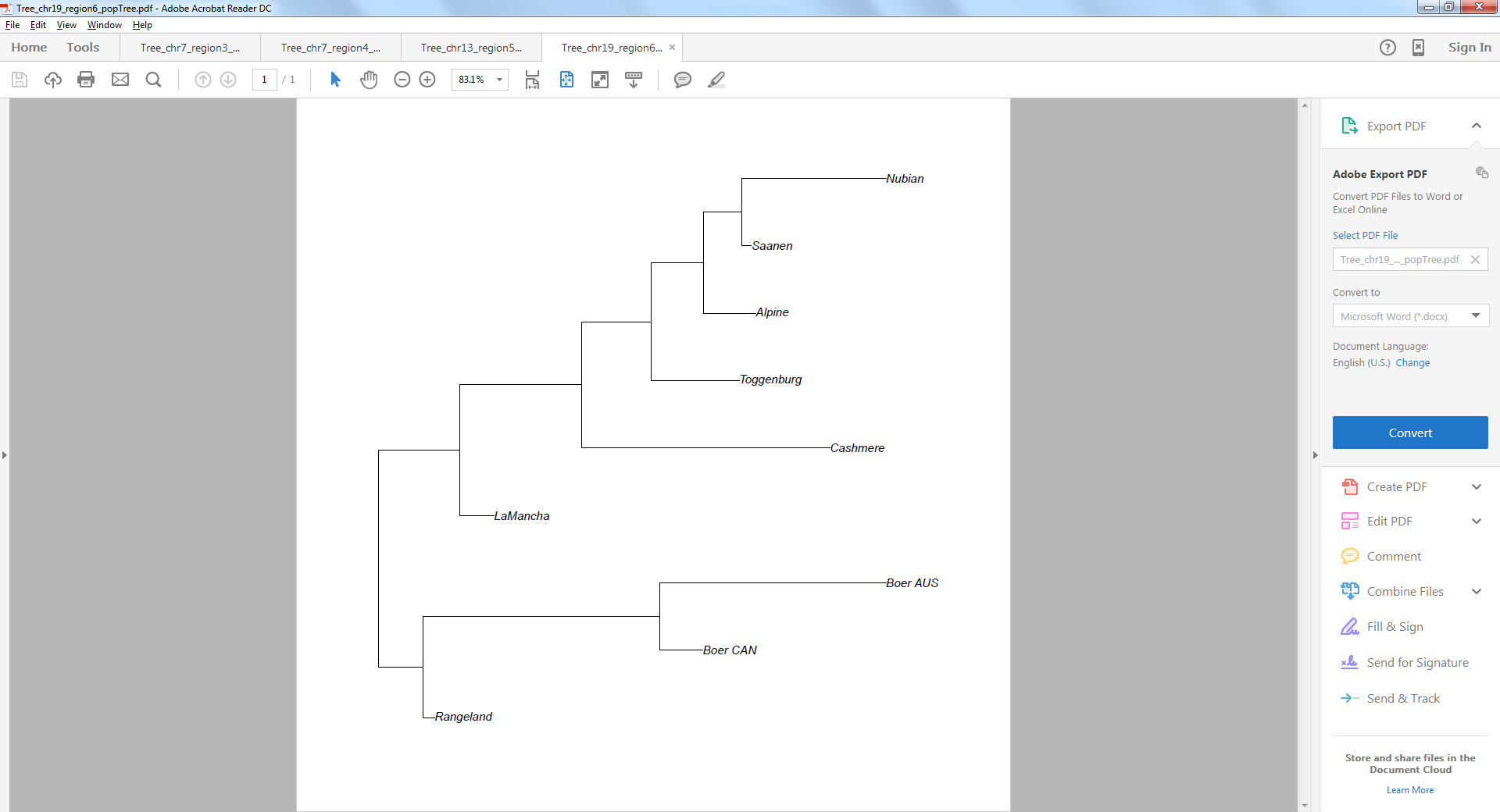


**Figure S6.** Population tree using significant SNPs for region 6, chromosome 19.


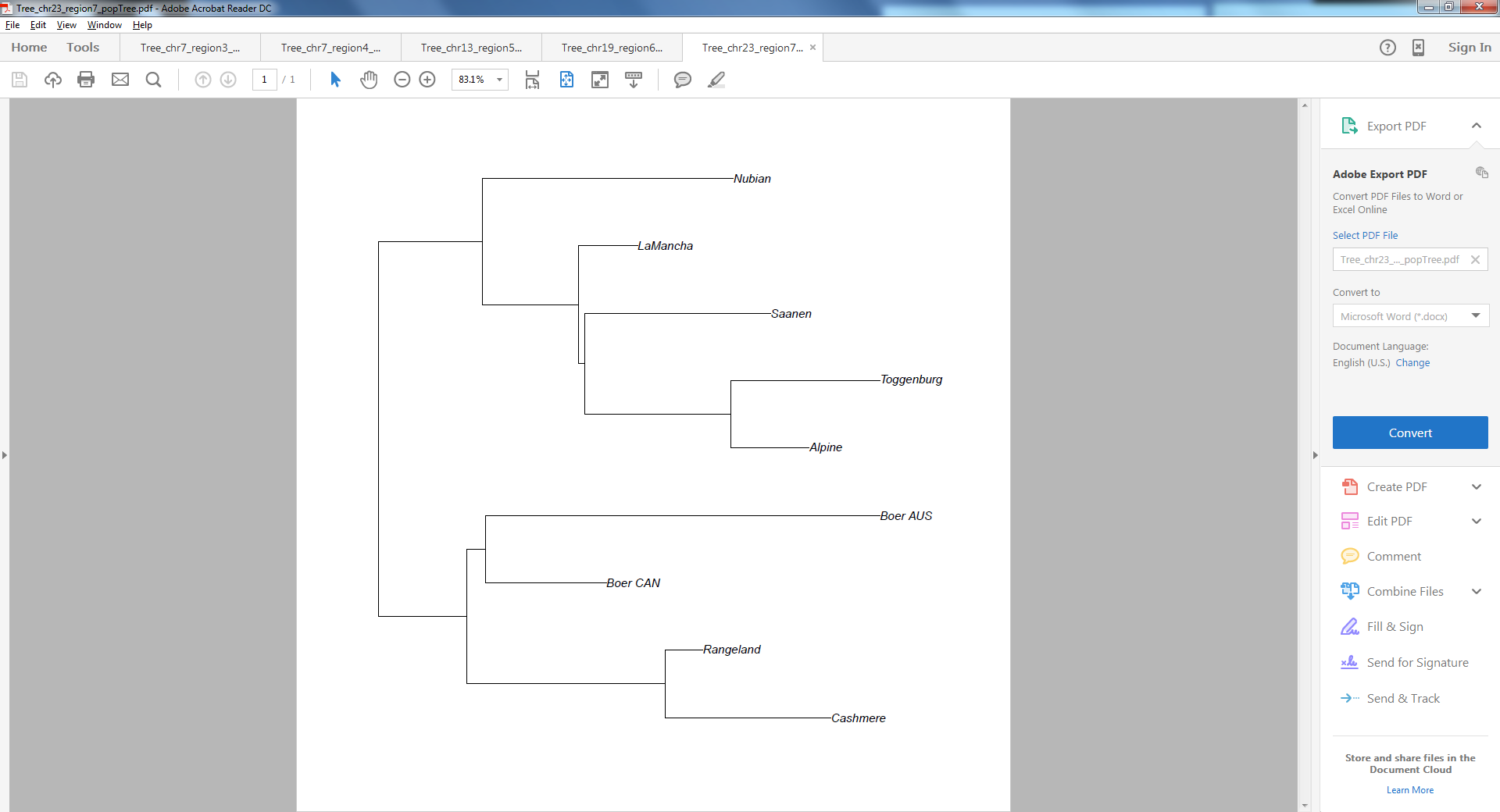


**Figure S7.** Population tree using significant SNPs for region 7, chromosome 23.
