# Supplementary material for: Genetic diversity and signatures of selection in various goat breeds revealed by genome-wide SNP markers
Source: BMC Genomics. 2017 Mar 14;18:229. doi: 10.1186/s12864-017-3610-0 (PMC5348779; doi:10.1186/s12864-017-3610-0)
Supplement: Additional file 9: — Panther plot of the biological pathways represented within genes located in each significant region identified in this study. (DOCX 101 kb) [file 12864_2017_3610_MOESM9_ESM.docx]

**Figure S1.** Panther plot of the biological pathways represented within genes located in the significant region of chromosome 10 for the Alpine breed in the scenario FST1.

**Figure S2.** Panther plot of the biological pathways represented within genes located in the significant region of chromosome 13 for the Australian Boer breed in the scenario FST1.

**Figure S3.** Panther plot of the biological pathways represented within genes located in the significant region of chromosome 3 for the Canadian Boer breed in the scenario FST1.

**Figure S4.** Panther plot of the biological pathways represented within genes located in the significant region of chromosome 20 for the Canadian Boer breed in the scenario FST1.

**Figure S5.** Panther plot of the biological pathways represented within genes located in the significant region of chromosome 24 for the Canadian Boer breed in the scenario FST1.

**Figure S6.** Panther plot of the biological pathways represented within genes located in the significant region of chromosome 7 for the LaMancha breed in the scenario FST1.

**Figure S7.** Panther plot of the biological pathways represented within genes located in the significant region of chromosome 6 for the Nubian breed in the scenario FST1.

**Figure S8.** Panther plot of the biological pathways represented within genes located in the significant region of chromosome 11 for the Nubian breed in the scenario FST1.

**Figure S9.** Panther plot of the biological pathways represented within genes located in the significant region of chromosome 22 for the Nubian breed in the scenario FST1.

**Figure S10.** Panther plot of the biological pathways represented within genes located in the significant region of chromosome 6 for the Rangeland breed in the scenario FST1.

**Figure S11.** Panther plot of the biological pathways represented within genes located in the significant region of chromosome 6 for the Saanen breed in the scenario FST1.

**Figure S12.** Panther plot of the biological pathways represented within genes located in the significant region of chromosome 3 for the Meat breeds group in the scenario FST2.

**Figure S13.** Panther plot of the biological pathways represented within genes located in the significant region of chromosome 13 for the Meat breeds group in the scenario FST2.

**Figure S14.** Panther plot of the biological pathways represented within genes located in the significant region of chromosome 20 for the Meat breeds group in the scenario FST2.

**Figure S15.** Panther plot of the biological pathways represented within genes located in the significant region of chromosome 6 for the Dual-purpose group in the scenario FST3.

**Figure S16.** Panther plot of the biological pathways represented within genes located in the significant region of chromosome 22 for the Dual-purpose group in the scenario FST3.

**Figure S17.** Panther plot of the biological pathways represented within genes located in the significant region of chromosome 3 for the Meat breeds group in the scenario FST3.

**Figure S18.** Panther plot of the biological pathways represented within genes located in the significant region of chromosome 6 for Meat breeds group in the scenario FST3.

**Figure S19.** Panther plot of the biological pathways represented within genes located in the significant region of chromosome 3 for the Meat breeds group in the scenario FST3.

**Figure S20.** Panther plot of the biological pathways represented within genes located in the significant region of chromosome 20 for the Meat breeds group in the scenario FST3.

**Figure S21.** Panther plot of the biological pathways represented within genes located in the significant region of chromosome 7 identified through hapFLK approach.

**Figure S22.** Panther plot of the biological pathways represented within genes located in the significant region of chromosome 7 (region 2) identified through hapFLK approach.

**Figure S23.** Panther plot of the biological pathways represented within genes located in the significant region of chromosome 19 identified through hapFLK approach.
